# Sex differences in the prevalence of neural tube defects and preventive effects of folic acid (FA) supplementation among five counties in northern China: results from a population-based birth defect surveillance programme

**DOI:** 10.1136/bmjopen-2018-022565

**Published:** 2018-11-08

**Authors:** Jufen Liu, Jing Xie, Zhiwen Li, Nicholas D E Greene, Aiguo Ren

**Affiliations:** 1 Institute of Reproductive and Child Health, Key Laboratory of Reproductive Health, National Health Commission of the People’s Republic of China, Peking University, Beijing, China; 2 Department of Epidemiology and Biostatistics, School of Public Health, Peking University, Beijing, China; 3 UCL Great Ormond Street Institute of Child Health, University College London, London, UK

**Keywords:** epidemiology, public health, neurogenetics

## Abstract

**Objectives:**

Sex differences in prevalence of neural tube defects (NTDs) have previously been recognised; however, the different susceptibility of men and women have not been examined in relation to the effects of folic acid (FA) supplementation. We hypothesised that FA may have a disproportionate effect that alters the sex-specific prevalence of NTDs.

**Setting:**

Data from two time points, before (2003–2004) and after (2011–2016) the start of the supplementation programme, were obtained from a population-based birth defect surveillance programme among five counties in northern China. All live births (28 or more complete gestational weeks), all stillbirths of at least 20 weeks’ gestational age and pregnancy terminations at any gestational age following the prenatal diagnosis of NTDs were included.

**Participants:**

A total of 25 249 and 83 996 births before and after the programme were included respectively.

**Primary and secondary outcome measures:**

The prevalence of NTDs by sex and subtype, Male:female rate ratios and their 95% CI were calculated.

**Results:**

Overall, NTDs were less prevalent among men than among women (rate ratio (RR) 0.92; 95% CI 0.90 to 0.94), so was anencephaly (RR 0.77; 95% CI 0.73 to 0.81) and encephalocele (RR 0.75; 95% CI 0.61 to 0.92), while spina bifida showed a male predominance (RR 1.10; 95% CI 1.05 to 1.15). The overall prevalence of NTDs decreased by 78/10 000 in men and 108.7/10 000 in women from 2003 to 2004 to 2011 to 2016. There was a significant sex difference in the magnitude of reduction, being greater in women than men, particularly for anencephaly.

**Conclusions:**

The prevalence of NTDs decreased in both sexes after the implementation of a massive FA supplementation programme. While female predominance was observed in open NTDs and total NTDs, they also had a greater rate of decrease in NTDs after the supplementation programme.

**Strengths and limitations of this study**

- Our data are from a population-based surveillance system, which covered all live births (28 or more complete gestational weeks), and neural tube defect (NTD) cases from pregnancies of any gestational age.
- NTDs decreased among both men and women after the implementation of a massive folic acid (FA) supplementation programme in China, while these decreases were significantly greater in women than in men.
- The reduction in NTD cases during the post-FA period inversely mirrors the increase in plasma folate concentrations among pregnant women.
- Limitations of our study include that covering small regions and lacking of detailed subtypes of spina bifida.

## Introduction

The prevalence of many types of birth defects appears to differ by sex, with the majority of them being more prevalent among men,^1 2^ including orofacial cleft (cleft lip with or without cleft palate), urinary system (hypospadias, hydronephrosis) and gastrointestinal tract (diaphragmatic hernia). However, some types of neural tube defects (NTDs) occur more frequently in women (eg, isolated cases of cranial defects, such as anencephaly).^3 4^ Women tend to have anencephaly more often than men, while craniorachischisis and spina bifida involving the higher spine may also show a preponderance among women.^4^ By contrast, there tends to be a higher proportion of men than women with spina bifida affecting the lower spine.^4^ There are thought to be multiple initiation sites in human neural tube closure,^5^ as described in other mammalian embryos. Neural tube closure occurs during weeks 3–4 of human gestation and the sex of the embryo is differentially associated with a lack of closure of specific areas of the neural tube.^5^

Female predisposition to anencephaly is also observed among a number of mouse models,^3 6^ but the mechanism underlying this has not been established. In mice, female embryos tend to be developmentally delayed compared with their male littermates at neurulation stages, leading to the hypothesis that the longer duration of cranial neural tube closure would lead to greater susceptibility to NTD-causing insult. However, going against this hypothesis, the delay in development may occur prior to neural tube closure, with female and male embryos taking the same time to complete cranial neurulation.^7^ Other suggested mechanisms include sex differences in genetic risk, epigenetic factors, a differential rate of fetal loss and susceptibility to environmental influences.^8^

Among environmental factors, periconceptional supplementation with folic acid (FA) has been shown to dramatically reduce the risk for NTDs in offspring^9^ and, conversely, suboptimal maternal folate status is a risk factor for NTDs.^10^ Although a large number of studies have investigated the preventive effects of FA on NTDs, the influence of maternal FA supplementation on sex-associated risk for NTDs and their subtypes is unclear. Some studies have lacked data on the type of NTDs or sex,^11^ while others have not found significant differences among subtypes of NTD.^12^ In these studies, the apparent protective effects of FA do not seem to act preferentially in one region of the neural tube and there appears to be a general reduction in the occurrence of human NTDs.^13^ In a study in Mexico in which weekly administration of 5 mg FA was associated with a 50% reduction in the incidence of NTDs, the main effect was on spina bifida, with a higher reduction in female cases.^8^ In several countries, FA fortification (FAF) of food has been associated with a decreased frequency of spina bifida and anencephaly on a population level.^14 15^ A recent study from South America showed that prevalence of NTDs, particularly anencephaly and cervicothoracic spina bifida, showed a greater reduction rate in women than in men after FAF resulting in a change of the sex ratio of infants with NTDs.^16^

In 2009, the Ministry of Health of China initiated a nationwide FA supplementation programme for the purpose of further decreasing the occurrence of NTDs in the country.^17^ Women with a rural household registration and planning to become pregnant are eligible to obtain FA supplements free of charge through the maternal healthcare system. In a recent study, we showed that birth prevalence of NTDs in the five counties of Shanxi Province in northern China dropped dramatically, from 118.9/10 000 in 2000 to 31.5/10 000 in 2014.^18^ In the present study, we examined the trends in NTD prevalence by sex in five counties of Shanxi Province, China, from 2003 to 2016, encompassing the introduction of a massive FA supplementation campaign. Characterisation of the sex distribution of NTDs may be helpful to better understand NTD development in association with FA supplementation.

## Materials and methods

### Birth defect surveillance

Five counties in Shanxi Province (Pingding, Xiyang, Taigu, Zezhou and Shouyang) were included in a population-based birth defects surveillance system in the current study. The surveillance system was similar to a previously reported system, created to meet the primary objective of monitoring the prevalence of major structural birth defects including NTDs.^19^ A national FA supplementation programme was initiated in China in 2009; therefore, in the current study, we collected data at two time points: before (2003–2004) and after (2011–2016) the start of the supplementation programme. All pregnant women who resided in the study area for more than 1 year during the period were monitored. All live births (28 or more complete gestational weeks), all stillbirths of at least 20 weeks’ gestational age and pregnancy terminations at any gestational age following the prenatal diagnosis of NTDs were included. Photographs of every suspected case were obtained. NTD diagnosis was conducted by local specialists in maternal-fetal medicine, and photographs were sent to a paediatrician at Peking University for confirmation. The study protocol was reviewed and approved.

### Statistical analysis

The prevalence of NTDs by infant sex was analysed separately for open NTDs (anencephaly, spina bifida)^20^ and encephalocele. The denominator was the total number of all births and pregnancy that reached 28 or more complete gestational weeks by sex; the numerator was the number of NTD cases regardless of gestational age by sex. The prevalence of NTDs by sex and subtype were compared using χ^2^ tests. A difference in differences (DID) model was applied to compare sex differences between the time periods.^21^ A two-tailed p≤0.05 was considered to indicate statistically significant differences. All statistical analyses were performed using the SPSS package V.20.0 (SPSS, Chicago, USA).

### Patient and public involvement

Our main analysis was based on the birth defects surveillance system; the information was anonymised. Patients and or public were not involved.

## Results

### Prevalence before FA supplementation programme

The prevalence of NTDs by subtype and sex is shown in table 1. Before the programme, a total of 25 249 births and 341 cases of NTDs (156 men, 185 women) were recorded in the system. The overall prevalence of NTDs for both sexes was 135.1/10 000, comprising 118.6/10 000 among men and 152.9/10 000 among women. All subtypes except spina bifida were more prevalent among women with a male-to-female rate ratio (RR) of 0.60 (95% CI 0.45 to 0.80) and 0.79 (95% CI 0.77 to 0.81) for encephalocele and open NTDs, respectively (table 1).

**Table 1:** Prevalence rates of NTDs (per 10 000 births) by type and sex in Shanxi Province, China, in 2003–2004 and 2011–2016

NTDs type	Male			Female			M:F rate ratio		
	Births	Cases	Rate	Births	Cases	Rate	RR	95% CI	
2003–2004 (Pre-FA supplementation)									
	13 150			12 099					
Open NTDs		145	110.3		168	138.9	0.79	0.77	0.81
Anencephaly		65	49.4		94	77.7	0.64	0.61	0.67
Spina bifida		80	60.8		74	61.2	0.99	0.94	1.04
Encephalocele		11	8.4		17	14.1	0.60	0.45	0.80
Total NTDs		156	118.6		185	152.9	0.78	0.76	0.80
2011–2016 (Post-FA supplementation)									
	43 538			40 458					
Open NTDs		160	36.7		158	39.1	0.94	0.92	0.96
Anencephaly		64	14.7		77	19.0	0.77	0.73	0.81
Spina bifida		96	22.0		81	20.0	1.10	1.05	1.15
Encephalocele		17	3.9		21	5.2	0.75	0.61	0.92
Total NTDs		177	40.7		179	44.2	0.92	0.90	0.94

NTDs, neural tube defects; RR, relative risk.

### Prevalence during FA supplementation programme

After the initiation of the programme, a total of 83 996 births and 356 cases of NTDs (177 men, 179 women) were recorded in the system. The overall prevalence for both sexes was 43.3/10 000, being 40.7/10 000 among men and 44.2/10 000 among women. Hence, in sum, NTDs were less prevalent among men than women (RR 0.92; 95% CI 0.90 to 0.94), as was anencephaly (RR 0.77; 95% CI 0.73 to 0.81) and encephalocele (RR 0.75; 95% CI 0.61 to 0.92), while spina bifida showed male predominance (RR 1.10; 95% CI 1.05 to 1.15) (table 1).

### Differences in changes in prevalence rates of NTDs

After the supplementation period, the prevalence of all types of NTDs was lower in both male and female fetuses. However, there was a significant sex difference in the magnitude of reduction, being more pronounced among women, especially for open NTDs including anencephaly (p<0.01). The overall prevalence of NTDs decreased by 78/10 000 in men and 108.7/10 000 in women in absolute terms; anencephaly decreased by 70.3% in men and 75.5% in women in relative term (table 2).

**Table 2:** Differences in changes in prevalence rates of NTDs (per 10 000 births) by type and sex in Shanxi Province, China, in 2003–2004 and 2011–2016

Types	Male rate		Female rate		Female–Male DID value	P values
	Absolute	Relative	Absolute	Relative		
Open NTDs	73.5	66.7	99.8	71.9	26.3	0.001
Anencephaly	34.7	70.3	58.7	75.5	23.9	0.001
Spina bifida	38.8	63.8	41.1	67.3	2.4	0.765
Encephalocele	4.5	53.3	8.9	63.1	4.4	0.213
Total NTDs	78.0	65.7	108.7	71.1	30.7	0.007

DID, difference in difference; NTDs, neural tube defects.

Absolute rate of decrease in prevalence=prevalence of group before folic acid supplementation–prevalence of group after folic acid supplementation.

Relative rate of decrease in prevalence=absolute rate of decrease in prevalence/prevalence of group before folic acid supplementation.

## Discussion

This quasi-experimental population-based study examined whether introduction of population-wide FA supplementation was associated with changes in the sex ratio for NTDs prevalence in northern China, as a means to understand whether the preventive effects of FA supplementation differ by sex. We found that, after the introduction of the national FA supplementation campaign, the prevalence of total NTDs and open NTDs (including anencephaly and spina bifida) decreased among both sexes. However, the magnitude of decline was significantly greater among women than men.

The prevalence of NTDs in Shanxi Province in northern China is historically high, being 105.5 per 10 000 births in the late 1980s^22^ and 138 per 10 000 births in 2003.^23^ In 2009, the Ministry of Health of China initiated a nationwide FA supplementation programme for the purpose of further decreasing the occurrence of NTDs in the country.^17^ Women with a rural household registration and planning to become pregnant are eligible to obtain FA supplements free of charge through the maternal healthcare system. In a recent study, we showed that birth prevalence of NTDs in the five counties of Shanxi Province in northern China dropped dramatically, from 118.9/10 000 in 2000 to 31.5/10 000 in 2014.^18^ However, differences between the sexes among this population have rarely been examined. One previous study reported a female predominance among anencephaly cases (male-to-female RR 0.49; 95% CI 0.30–0.79) but not among spina bifida (RR 0.90; 95% CI 0.55 to 1.45) or encephalocele (RR 1.03; 95% CI 0.40 to 2.69) cases in 2006^23^; however, that study was conducted before the national FA supplementation programme and how the NTD prevalence has changed in each sex in association with the programme remains unknown.

Since 2009, the nationwide FA supplementation programme has provided FA supplements, free of charge, to all women who have a rural registration and who plan to become pregnant. As we previously reported, the prevalence of NTDs was dramatically lower in 2012–2014 than in 2009 in Shanxi Province. This decrease may be partially attributable to the programme; however, this decreasing trend has not continued since 2014. In fact, the prevalence in the same area was higher in 2015–2016 (32.8 per 10 000 births) than in 2014 (31.5 per 10 000 births). Moreover, the prevalence of NTDs in this area remains high when compared with the rates in the USA,^15^ Canada,^24^ Germany,^25^ England, Wales^26^and Western Australia.^27^ The compliance of women taking the FA pills may be attributable to the high prevalence. A previous study showed the percentage of folic acid supplementation before the last menstrual period increased only from 30% to 43% and the supplementation adherence of ≥8 days/10 days showed a decrease.^28^ Other reasons may need further study.

In addition to the NTD prevalence data, our previous cross-sectional surveys on the use of FA and blood folate concentration among pregnant women provide external evidence to test the correlation between folate status and NTDs. Those two surveys took place in the same area, one before the FA programme started (2003–2004)^29^ and the other after it started (2011–2012).^30^ Detailed descriptions of the two studies have already been published.^30 31^ Briefly, a face-to-face interview with a structured questionnaire including information on FA supplementation was conducted among women who was in their late first trimester or early second trimester of pregnancy. A 5 mL non-fasting blood sample was collected at the time of recruitment and plasma levels of folate were determined using a microbiological assay.^32^

Our previous cross-sectional study showed that the proportion of people taking FA supplements increased from 9.2% in 2003–2004^33^ to 66.3% in 2011–2012^30^. Comparison of population data on blood folate concentration among pregnant women and NTD prevalence data indicated a clear trend: plasma folate concentration increased from 10.4 nmol/L in 2003–2004^33^ to 33.4 nmol/L in 2011–2012^22^ while the prevalence of NTDs decreased from 117.8/10 000 births to 60.3/10 000 births, respectively.^19^ Women who took FA supplements had significantly higher folate concentrations (median 41.9 nmol/L) than those who did not (13.2 nmol/L) in 2003–2004. In contrast, these values were 50.7 nmol/L and 17.3 nmol/L, respectively, in 2011–2012. The prevalence of NTDs was lower among both men and women after the programme. However, the effect was not the same for men and women, being greater among women than men. For open NTDs, the decrease was 26.3/10 000 higher among women than men; for total NTDs (including encephalocele), the decrease was 30.7/10 000 higher among women than men in the current study.

Studies on mice have indicated that female embryos are more susceptible to NTDs than males in some, but not all, genetic models.^34^ For example, there is a higher rate of cranial NTDs in mice carrying mutations in *Trp53*,^6^ *Pax3*, or *Nf1*^35^ but not *Gldc*.^36^ Sex differences in NTD susceptibility in *Trp53* null mice may result from the presence of two X chromosomes and independent of the Y chromosome.^6^ Given the association between abnormal one-carbon metabolism or impaired methylation and NTDs, it has been hypothesised that the higher rate of cranial NTDs in females may be an epigenetic phenomenon. The demand for genomic DNA methylation is higher in females, which methylate most of the DNA in the large inactive X chromosome after every cell division. It is speculated that this may reduce the methylation capacity available for other key cellular needs.^3^ In the splotch (*Sp^2H^*; *Pax3* mutant) strain, female susceptibility to exencephaly is exacerbated by dietary folate deficiency to a greater extent than in males.^37^ While the aetiology of human NTDs is more complex than in single-gene models, if such a scenario occurs in some human NTDs, then it might be predicted that an overall increase in population-level folate status may have a greater effect on NTDs in women than in men. This is consistent with our findings for anencephaly rates.

Genetic and environmental factors may also interact to determine murine sex ratios, with female embryos being more susceptible to lethality. In the splotch strain, for example, arsenite treatment and advanced maternal age are both associated with higher rates of in utero death and NTDs.^38^ Both factors result in elevated sex ratios (male:female) among litters, which suggests increased loss of female embryos by resorption. This effect is genotype-dependent such that a greater effect, with lower sex ratio, is noted as the number of *Sp*(*Pax3* mutant) alleles increases.

A study of human NTDs indicated an overall excess of affected women, with the overall male:female sex distribution of unaffected carrier or transmitting individuals being 0.64, and an even greater excess of female gene carriers, with a male:female ratio of 0.52, when only closely related relatives are counted.^39^ To fully understand the genetic influences of NTDs, future studies are needed to investigate the importance of epigenetic factors and imprinting effects, methylation status and folate supplementation, as well as the gender of the child in altering NTD risk.

In fact, the larger decline in NTD prevalence among women is somewhat expected given that it was higher in the presupplementation time period. The difference between sexes which is less pronounced is consistent with prevalences beginning to converge toward the prevalence of NTDs that are not preventable by FA.

Our data are from a population-based surveillance system, which covered all live births (28 or more complete gestational weeks), and NTD cases from pregnancies of any gestational age. This could give a more precise estimation of NTD rate than hospital-based surveillance systems that include only NTD cases in pregnancies of 28 gestational weeks or greater in China.^40^

Three main limitations of our study should be mentioned. First, we included data from only five counties, and the results may not be generalisable to the entire province or country. Second, we did not include the detailed subtypes of spina bifida, such as high-level and low-level lesions, which may show different patterns. A future study may be useful to address this question. Third, as an ecological design in which some confounding factors might affect our results, the massive FA supplementation programme was the only major factor that may affect the population rate of NTDs in the study. Improvement in living standards and prenatal diagnosis may also impact the rate of NTDs, but these potential factors are not predicted to have a differential effect on men and women.

In conclusion, the prevalence of NTDs decreased in both sexes after the implementation of a massive FA supplementation programme, but to a greater degree (both total NTDs and open NTDs) in women.

## Supplementary Material

Reviewer comments

Author's manuscript

## References

[1] Hay S Sex differences in the incidence of certain congenital malformations: a review of the literature and some new data. Teratology 1971;4:277–86. 10.1002/tera.1420040303 5568039

[2] Michalski AM, Richardson SD, Browne ML, et al Sex ratios among infants with birth defects, National Birth Defects Prevention Study, 1997-2009. Am J Med Genet A 2015;167A:1071–81. 10.1002/ajmg.a.36865 25711982

[3] Juriloff DM, Harris MJ Hypothesis: the female excess in cranial neural tube defects reflects an epigenetic drag of the inactivating X chromosome on the molecular mechanisms of neural fold elevation. Birth Defects Res A Clin Mol Teratol 2012;94:849–55. 10.1002/bdra.23036 22753363

[4] Tennant PW, Samarasekera SD, Pless-Mulloli T, et al Sex differences in the prevalence of congenital anomalies: a population-based study. Birth Defects Res A Clin Mol Teratol 2011;91:894–901. 10.1002/bdra.22846 21987467

[5] Seller MJ Sex, neural tube defects, and multisite closure of the human neural tube. Am J Med Genet 1995;58:332–6. 10.1002/ajmg.1320580406 8533841

[6] Chen X, Watkins R, Delot E, et al Sex difference in neural tube defects in p53-null mice is caused by differences in the complement of X not Y genes. Dev Neurobiol 2008;68:265–73. 10.1002/dneu.20581 18004765

[7] Brook FA, Estibeiro JP, Copp AJ Female predisposition to cranial neural tube defects is not because of a difference between the sexes in the rate of embryonic growth or development during neurulation. J Med Genet 1994;31:383–7. 10.1136/jmg.31.5.383 8064816 PMC1049870

[8] Martínez de Villarreal LE, Arredondo P, Hernández R, et al Weekly administration of folic acid and epidemiology of neural tube defects. Matern Child Health J 2006;10:397–401. 10.1007/s10995-006-0082-2 16900407

[9] Berry RJ, Li Z, Erickson JD, et al Prevention of neural-tube defects with folic acid in China. China-U.S. Collaborative project for neural tube defect prevention. N Engl J Med 1999;341:1485–90. 10.1056/NEJM199911113412001 10559448

[10] Crider KS, Devine O, Hao L, et al Population red blood cell folate concentrations for prevention of neural tube defects: Bayesian model. BMJ 2014;349:g4554 10.1136/bmj.g4554 25073783 PMC4115151

[11] Smithells RW, Sheppard S, Schorah CJ, et al Apparent prevention of neural tube defects by periconceptional vitamin supplementation. 1981. Int J Epidemiol 2011;40:1146–54. 10.1093/ije/dyr143 22039189

[12] MRC Vitamin Study Research Group. Prevention of neural tube defects: results of the medical research council vitamin study. The Lancet 1991;338:131–7. 10.1016/0140-6736(91)90133-A 1677062

[13] Seller MJ Multi-site neural tube closure in humans and maternal folate supplementation. Am J Med Genet 1995;58:222–4. 10.1002/ajmg.1320580305 8533821

[14] Honein MA, Paulozzi LJ, Mathews TJ, et al Impact of folic acid fortification of the US food supply on the occurrence of neural tube defects. JAMA 2001;285:2981–6. 10.1001/jama.285.23.2981 11410096

[15] Williams J, Mai CT, Mulinare J, et al Updated estimates of neural tube defects prevented by mandatory folic acid fortification - United States, 1995-2011. MMWR Morb Mortal Wkly Rep 2015;64:1–5. 25590678 PMC4584791

[16] Poletta FA, Rittler M, Saleme C, et al Neural tube defects: sex ratio changes after fortification with folic acid. PLoS One 2018;13:e0193127 10.1371/journal.pone.0193127 29538416 PMC5851584

[17] Ren AG Prevention of neural tube defects with folic acid: the Chinese experience. World J Clin Pediatr 2015;4:41–4. 10.5409/wjcp.v4.i3.41 26261765 PMC4526837

[18] Liu J, Zhang L, Li Z, et al Prevalence and trend of neural tube defects in five counties in shanxi province of northern China, 2000 to 2014. Birth Defects Res A Clin Mol Teratol 2016;106:267–74. 10.1002/bdra.23486 26879384

[19] Li S, Moore CA, Li Z, et al A population-based birth defects surveillance system in the people’s republic of China. Paediatr Perinat Epidemiol 2003;17:287–93. 10.1046/j.1365-3016.2003.00478.x 12839541

[20] McComb JG A practical clinical classification of spinal neural tube defects. Childs Nerv Syst 2015;31:1641–57. 10.1007/s00381-015-2845-9 26351218

[21] Ashenfelter O, Card D Using the longitudinal structure of earnings to estimate the effect of training programs. Rev Econ Stat 1985;67:648–60. 10.2307/1924810

[22] Xiao KZ, Zhang ZY, Su YM, et al Central nervous system congenital malformations, especially neural tube defects in 29 provinces, metropolitan cities and autonomous regions of china: Chinese birth defects monitoring program. Int J Epidemiol 1990;19:978–82. 2084031 10.1093/ije/19.4.978

[23] Li Z, Ren A, Zhang L, et al Extremely high prevalence of neural tube defects in a 4-county area in shanxi province, China. Birth Defects Res A Clin Mol Teratol 2006;76:237–40. 10.1002/bdra.20248 16575897

[24] De Wals P, Tairou F, Van Allen MI, et al Reduction in neural-tube defects after folic acid fortification in Canada. N Engl J Med 2007;357:135–42. 10.1056/NEJMoa067103 17625125

[25] Nasri K, Ben Fradj MK, Hamdi T, et al Epidemiology of neural tube defect subtypes in Tunisia, 1991-2011. Pathol Res Pract 2014;210:944–52. 10.1016/j.prp.2014.06.027 25110062

[26] Morris JK, Wald NJ Prevalence of neural tube defect pregnancies in England and Wales from 1964 to 2004. J Med Screen 2007;14:55–9. 10.1258/096914107781261945 17626701

[27] Bower C, D’Antoine H, Stanley FJ Neural tube defects in Australia: trends in encephaloceles and other neural tube defects before and after promotion of folic acid supplementation and voluntary food fortification. Birth Defects Res A Clin Mol Teratol 2009;85:269–73. 10.1002/bdra.20536 19180646

[28] Liu J, Jin L, Meng Q, et al Changes in folic acid supplementation behaviour among women of reproductive age after the implementation of a massive supplementation programme in China. Public Health Nutr 2015;18:582–8. 10.1017/S1368980014000950 24844654 PMC10271069

[29] Ren A, Zhang L, Hao L, et al Comparison of blood folate levels among pregnant Chinese women in areas with high and low prevalence of neural tube defects. Public Health Nutr 2007;10:762–8. 10.1017/S1368980007246786 17381897

[30] Liu J, Gao L, Zhang Y, et al Plasma folate levels in early to mid pregnancy after a nation-wide folic acid supplementation program in areas with high and low prevalence of neural tube defects in China. Birth Defects Res A Clin Mol Teratol 2015;103:501–8. 10.1002/bdra.23368 25808729

[31] Ren A, Zhang L, Li Z, et al Awareness and use of folic acid, and blood folate concentrations among pregnant women in northern China–an area with a high prevalence of neural tube defects. Reprod Toxicol 2006;22:431–6. 10.1016/j.reprotox.2006.02.003 16549319

[32] O’Broin S, Kelleher B Microbiological assay on microtitre plates of folate in serum and red cells. J Clin Pathol 1992;45:344–7. 10.1136/jcp.45.4.344 1577973 PMC495277

[33] Zhang L, Ren A, Li Z, et al Folate concentrations and folic acid supplementation among women in their first trimester of pregnancy in a rural area with a high prevalence of neural tube defects in Shanxi, China. Birth Defects Res A Clin Mol Teratol 2006;76:461–6. 10.1002/bdra.20271 16933216

[34] Copp AJ, Stanier P, Greene ND Neural tube defects: recent advances, unsolved questions, and controversies. Lancet Neurol 2013;12:799–810. 10.1016/S1474-4422(13)70110-8 23790957 PMC4023229

[35] Lakkis MM, Golden JA, O’Shea KS, et al Neurofibromin deficiency in mice causes exencephaly and is a modifier for splotch neural tube defects. Dev Biol 1999;212:80–92. 10.1006/dbio.1999.9327 10419687

[36] Pai YJ, Leung KY, Savery D, et al Glycine decarboxylase deficiency causes neural tube defects and features of non-ketotic hyperglycinemia in mice. Nat Commun 2015;6:6388 10.1038/ncomms7388 25736695 PMC4366506

[37] Burren KA, Savery D, Massa V, et al Gene-environment interactions in the causation of neural tube defects: folate deficiency increases susceptibility conferred by loss of Pax3 function. Hum Mol Genet 2008;17:3675–85. 10.1093/hmg/ddn262 18753144 PMC2581426

[38] Martin LJ, Machado AF, Loza MA, et al Effect of arsenite, maternal age, and embryonic sex on spina bifida, exencephaly, and resorption rates in the splotch mouse. Birth Defects Res A Clin Mol Teratol 2003;67:231–9. 10.1002/bdra.10006 12854658

[39] Deak KL, Siegel DG, George TM, et al Further evidence for a maternal genetic effect and a sex-influenced effect contributing to risk for human neural tube defects. Birth Defects Res A Clin Mol Teratol 2008;82:662–9. 10.1002/bdra.20511 18937341 PMC2981339

[40] Li K, He XB, Zhang L, et al [Impact of pregnancy terminations before 28 weeks of gestation on the overall prevalence of neural tube defects in two counties of Shanxi province]. Zhonghua Liu Xing Bing Xue Za Zhi 2012;33:509–12. 22883180

