# Supplementary material for: Sex differences in the prevalence of neural tube defects and preventive effects of folic acid (FA) supplementation among five counties in northern China: results from a population-based birth defect surveillance programme
Source: BMJ Open. 2018 Nov 8;8(11):e022565. doi: 10.1136/bmjopen-2018-022565 (PMC6231556; doi:10.1136/bmjopen-2018-022565)
Supplement: Author's manuscript [file bmjopen-2018-022565.draft_revisions.pdf]

BMJ Open is committed to open peer review. As part of this commitment we make the peer review history of every article we publish publicly available.

When an article is published we post the peer reviewers' comments and the authors' responses online. We also post the versions of the paper that were used during peer review. These are the versions that the peer review comments apply to.

The versions of the paper that follow are the versions that were submitted during the peer review process. They are not the versions of record or the final published versions. They should not be cited or distributed as the published version of this manuscript.

BMJ Open is an open access journal and the full, final, typeset and author-corrected version of record of the manuscript is available on our site with no access controls, subscription charges or pay-per-view fees (http://bmjopen.bmj.com).

If you have any questions on BMJ Open's open peer review process please email info.bmjopen@bmj.com

BMJ Open

Sex differences in the prevalence of neural tube defects and preventive effects of folic acid (FA) supplementation in northern China: Results from pre- and post-FA supplementation

Journal:	*BMJ Open*
Manuscript ID	bmjopen-2018-022565
Article Type:	Research
Date Submitted by the Author:	01-Mar-2018

Complete List of Authors:	Liu, Jufen; Peking University, Institute of Reproductive and Child Health<br>Xie, Jing; Peking University, Institute of Reproductive and Child Health<br>Li, Zhiwen; Peking University, Institute of Reproductive and Child Health<br>Greene, ND; University College London Institute of Child Health, Birth Defects Research Centre<br>Ren, Aiguo; Peking University, Institute of Reproductive & Child Health

Keywords:	EPIDEMIOLOGY, PUBLIC HEALTH, Neurogenetics < NEUROLOGY

SCHOLARONE™
Manuscripts

For peer review only

Title page

Title:

Sex differences in the prevalence of neural tube defects and preventive effects of folic acid (FA) supplementation in northern China: Results from pre- and post-FA supplementation

Names of authors:

Jufen Liu, Ph.D. [1,2], Jing Xie, BD [1,2], Zhiwen Li, Ph.D. [1,2*], Nicholas D. E. Greene , Ph.D.[3], Aiguo Ren, Ph.D.[1,2*]

Affiliations of authors:

[1] Institute of Reproductive and Child Health / Key Laboratory of Reproductive Health, National Health and Family Planning Commission of the People's Republic of China, Peking University, Beijing 100191, P. R. China

[2] Department of Epidemiology and Biostatistics, School of Public Health, Peking University, Beijing 100191, P. R. China

[3] UCL Great Ormond Street Institute of Child Health, University College London, London WC1N 1EH, United Kingdom

[*] Zhiwen Li and Aiguo Ren contributed equally to this paper

[*]**Corresponding author:**

Zhiwen Li, 38 College Rd, Haidian District, Institute of Reproductive and Child Health, Peking University Health Science Center, Beijing 100191, China. Tel.: +86-10-82801140; Fax: +86-10-82801141. Email: lizw@bjmu.edu.cn

Running head: Sex differences in NTDs in northern China

Word counts: 2185

For peer review only - http://bmjopen.bmj.com/site/about/guidelines.xhtml

Abstract

Objectives: Few population-based studies have examined sex differences among infants with NTDs in relation to the effects of folic acid (FA) supplementation. We hypothesized that FA may have a disproportionate effect that alters the sex-specific prevalence of NTDs.

Setting: Data from two time points, before (2003–2004) and after (2011–2016) the start of the supplementation program, were obtained from a population-based birth defect surveillance program among five counties in northern China. All pregnancies that reached 28 complete gestational weeks, including live births, stillbirths, and pregnancy terminations, and all NTDs regardless of gestational weeks were recorded.

Participants: A total of 25,249 and 82,258 births before and after the program were included respectively.

Primary and secondary outcome measures: The prevalence of NTDs by sex and subtype, Male:female sex ratios and their 95% confidence intervals were calculated.

Results: Overall, NTDs were less prevalent among males than among females (relative risk [RR], 0.92; 95% confidence interval [CI], 0.90–0.94), so was anencephaly (RR, 0.77; 95% CI, 0.73–0.81) and encephalocele (RR, 0.75; 95% CI, 0.61–0.92), while spina bifida showed a male predominance (RR, 1.10; 95% CI, 1.05–1.15). The overall prevalence of NTDs decreased by 78/10,000 in males and 108.7/10,000 in females from 2003–2004 to 2011–2016. There was a significant sex difference in the magnitude of reduction, being greater in females than males, particularly for anencephaly.

Conclusions: The prevalence of NTDs decreased in both sexes after the implementation of a massive FA supplementation program. While female predominance was observed in open NTDs and total NTDs, they also had a greater rate of decrease in NTDs after the supplementation program.

Key words: Sex differences, neural tube defects, folic acid supplementation

For peer review only - http://bmjopen.bmj.com/site/about/guidelines.xhtml

Strengths and limitations of this study

Neural tube defects are known to show sex differences in frequency, with anencephaly more common among female than male fetuses. Few population-based studies have examined sex differences among infants with neural tube defects in relation to the effect of folic acid (FA) supplementation. Using data from a population-based birth defect surveillance before and after implementation of a massive FA supplementation programme in northern China, this study presents estimates of the sex ratio for all NTDs and the specific subtypes.

- The prevalence of NTDs decreased for both sexes after the implementation of a massive FA supplementation program.
- There was a significant sex difference in the magnitude of reduction, the proportion of prevalence decrease was more pronounced among females than males.
- Our data are from a population-based surveillance system which could give a more precise estimation of NTDs than hospital-based surveillance systems that include only live births, as the majority of fetuses with NTDs are terminated following prenatal diagnosis before 28 gestational weeks in China.
- Comparison of population data on blood folate concentration among pregnant women and NTD prevalence data indicated a clear trend: plasma folate concentration increased while the prevalence of NTDs decreased.

For peer review only - http://bmjopen.bmj.com/site/about/guidelines.xhtml

INTRODUCTION

The prevalence of many types of birth defects appear to differ by sex, with the majority of them being more prevalent among males,[1,] including defects of the sex organs, urinary tract, and gastrointestinal tract. However, some types of neural tube defects (NTDs) occur more frequently in females (e.g., isolated cases of cranial defects).[2] There are multiple initiation sites in human neural tube closure,[3] as described in other mammalian embryos. Neural tube closure occurs during weeks 3–4 of human gestation and the sex of the embryo is differentially associated with a lack of closure of specific areas of the neural tube. Females tend to have anencephaly more often than males, while craniorachischisis and spina bifida involving the higher spine may also show a preponderance among females. By contrast, there tends to be a higher proportion of males than females with spina bifida affecting the lower spine.[4] Female predisposition to anencephaly is also observed among a number of mouse models.[5, 6] The mechanism underlying this has not been established. In mice, female embryos tend to be developmentally delayed compared to their male littermates at neurulation stages, leading to the hypothesis that the longer duration of cranial neural tube closure would lead to greater susceptibility to NTD-causing insult. However, going against this hypothesis, the delay in development may occur prior to neural tube closure, with female and male embryos taking the same time to complete cranial neurulation.[7] Other suggested mechanisms include sex differences in genetic risk, epigenetic factors, a differential rate of fetal loss, and susceptibility to environmental influences.[8]

Among environmental factors, periconceptional supplementation with FA has been shown to dramatically reduce the risk for NTDs in offspring[9] and conversely, suboptimal maternal folate status is a risk factor for NTDs. Although a large number of studies have investigated the preventive effects of folic acid (FA) on NTDs, the influence of maternal FA supplementation on sex-associated risk for NTDs and their subtypes is unclear. Some studies have lacked data on the type of NTDs or sex,[10]

For peer review only - http://bmjopen.bmj.com/site/about/guidelines.xhtml

others have not found significant differences among subtypes of NTD,[11] and in other studies the apparent protective effects of folate appear to be generalized.[12] In a study in Mexico in which weekly administration of 5 mg FA was associated with a 50% reduction in the incidence of NTDs, the main effect was on spina bifida, with a higher reduction in female cases.[8] Some studies have found that FA fortification of food is associated with a decreased frequency of spina bifida on a population level while anencephaly rate shows no decrease or is inconclusive.[13, 14]

The prevalence of NTDs in Shanxi Province in northern China is historically high, being 105.5 per 10,000 births in the late 1980s,[15] 138 per 10,000 births in 2003,[16] and 92.6 per 10,000 births in 2004–2010.[17] In 2009, the Ministry of Health of China initiated a nationwide FA supplementation program for the purpose of further decreasing the occurrence of NTDs in the country.[18] Women with a household registration and planning to become pregnant are eligible to obtain FA supplements free of charge through the maternal health care system. In a recent study, we showed that birth prevalence of NTDs in the five counties of Shanxi Province in northern China dropped dramatically, from 118.9/10,000 in 2000 to 31.5/10,000 in 2014.[19] However, differences between the sexes among this population have rarely been examined. One previous study reported a female predominance among anencephaly cases (male-to-female relative risk [RR], 0.49; 95% confidence interval [CI], 0.30–0.79) but not among spina bifida (RR, 0.90; 95% CI, 0.55–1.45) or encephalocele (RR, 1.03; 95% CI, 0.40–2.69) cases in 2006;[16] however, that study was conducted before the national FA supplementation program and how the NTD prevalence has changed in each sex in association with the program remains unknown.

Therefore, in the present study, we examined the trends in NTD prevalence by sex in five counties of Shanxi Province, China, from 2003 to 2016, encompassing the introduction of the massive FA supplementation campaign. Characterization of the sex distribution of NTDs may be helpful to provide personalized prevention recommendations for NTDs, and to better understand NTD development in

For peer review only - http://bmjopen.bmj.com/site/about/guidelines.xhtml

association with FA supplementation.

MATERIALS AND METHODS

Birth defect surveillance

Five counties in Shanxi Province (Pingding, Xiyang, Taigu, Zezhou, and Shouyang) were included in a population-based birth defects surveillance system in the current study. The surveillance system was similar to a previously reported system, created to meet the primary objective of monitoring the prevalence of major structural birth defects including NTDs.[20] A national FA supplementation program was initiated in China in 2009; therefore, in the current study we collected data at two time points: before (2003–2004) and after (2011–2016) the start of the supplementation program. All pregnant women who resided in the study area for more than 1 year during the period were monitored. All live births (28 or more complete gestational weeks), all stillbirths of at least 20 weeks' gestational age, and pregnancy terminations at any gestational age following the prenatal diagnosis of NTDs were included. Photographs of every suspected case were obtained. NTD diagnosis was conducted by local specialists in maternal-fetal medicine, and photographs were sent to a pediatrician at Peking University for confirmation. In addition to the NTD prevalence data, we also obtained cross-sectional survey data on the use of FA and blood folate concentration among pregnant women in the two periods, to provide external evidence to test the correlation between folate and NTDs. Those two surveys took place in the same area, one before the FA program started (2003–2004)[21] and the other after it started (2011–2012).[22] Plasma levels of folate were determined using a microbiological assay.[23] Detailed descriptions of the two studies have already been published.[21–22] Our study protocol was reviewed and approved by the Institutional Review Board of Peking University.

Statistical analysis

The prevalence of NTDs by infant sex was analyzed separately for open NTDs (anencephaly, spina bifida) and encephaloecele.[19] The denominator was the total

For peer review only - http://bmjopen.bmj.com/site/about/guidelines.xhtml

number of all births and pregnancy that reached 28 or more complete gestational weeks; the numerator was the number of NTDs cases regardless of gestational age. The prevalences of NTDs by sex and subtype were compared using Chi-square tests. A difference in differences (DID) model was applied to compare sex differences between the time periods.[24] A two-tailed $p \leq 0.05$ was considered to indicate statistically significant differences. All statistical analyses were performed using the SPSS package version 20.0 (SPSS Inc., Chicago, USA).

Patient and Public Involvement

Our main analysis was based on the birth defects surveillance system, the information was anonymized. Patients and or public were not involved.

RESULTS

Prevalence Before FA Supplementation Program

The prevalence of NTDs by subtype and sex are shown in Table 1. Before the program, a total of 25,249 births and 341 cases of NTDs (156 males, 185 females) were recorded in the system. The overall prevalence of NTDs for both sexes was 135.1/10,000, and it's 118.6/10,000 among males and 152.9/10,000 among females. All subtypes were more prevalent among females with a male-to-female RR of 0.60 (95% CI, 0.45–0.80) and 0.79 (95% CI, 0.77–0.81) for total NTDs and open NTDs, respectively (Table 1).

Prevalence After FA Supplementation Program

After the program, a total of 82,258 births and 356 cases of NTDs (177 males, 179 females) were recorded in the system. The overall prevalence for both sexes was 43.3/10,000, being 40.7/10,000 among males and 44.2/10,000 among females. Hence, in sum, NTDs were less prevalent among males than females (RR, 0.92; 95% CI, 0.90–0.94), so was anencephaly (RR, 0.77; 95% CI, 0.73–0.81) and encephalocele

For peer review only - http://bmjopen.bmj.com/site/about/guidelines.xhtml

(RR, 0.75; 95% CI, 0.61–0.92), while spina bifida showed male predominance (RR, 1.10; 95% CI, 1.05–1.15) (Table 1).

Differences in Changes in Prevalence Rates of NTDs

After the supplementation period, the prevalences of all types of NTDs were lower in both male and female fetuses. However, there was a significant sex difference in the magnitude of reduction, being more pronounced among females, especially for open NTDs including anencephaly ($p<0.01$). The overall prevalence of NTDs decreased by 78/10,000 in males and 108.7/10,000 in females; anencephaly decreased by 34.7% in males and 58.7% in females (Table 2).

NTD Prevalence in Association with Blood Folate

Comparison of population data on blood folate concentration among pregnant women and NTD prevalence data indicated a clear trend: plasma folate concentration increased from 10.4 nmol/L in 2002–2004[25] to 33.4 nmol/L in 2011–2012[22] while the prevalence of NTDs decreased from 117.8/10,000 births to 60.3/10,000 births, respectively[19] (Figure 1). Groups who reported taking FA supplements had significantly higher folate concentrations (median 41.9 nmol/L) than those who did not take FA (13.2 nmol/L) during the two time periods. In contrast, these values were 50.7 nmol/L and 17.3 nmol/L, respectively, in 2011–2012 (Figure 2).

DISCUSSION

This quasi-experimental population-based study examined whether population-wide FA supplementation was associated with changes in the sex bias among NTDs in northern China, as a means to understand whether the preventive effects of FA supplementation differ by sex. We found that, after the introduction of the national FA supplementation campaign, the prevalence of total NTDs and open NTDs (including anencephaly, spina bifida) decreased more significantly among females than males.

Since 2009, the nationwide FA supplementation program has provided FA

For peer review only - http://bmjopen.bmj.com/site/about/guidelines.xhtml

supplements, free of charge, to all women who have a rural registration and who plan to become pregnant. As we previously reported, the prevalence of NTDs was dramatically lower in 2012–2014 than in 2009 in Shanxi Province. This decrease may be partially attributable to the program; however, this decreasing trend has not continued since 2014. In fact, the prevalence in the same area was higher in 2016 than in 2014 (31.5 per 10,000 births). Moreover, the prevalence of NTDs in this area remains higher than in the USA,[26] Canada,[27] Germany,[28] England, Wales,[29] and Western Australia.[30]

The prevalence of NTDs was lower among both males and females after the program. FA supplementation was successful in that the proportion of people taking FA supplements increased from 9.2% in 2002–2004[6] to 66.3% in 2011–2012[22] in Shanxi Province, plasma folate concentrations were significantly higher after the program, and NTD prevalence was significantly lower. However, the effect was not the same for males and females, being greater among females than males. For open NTDs, the decrease was 26.3/10,000 higher among females than males; for total NTDs (including encephalocele), the decrease was 30.7/10,000 higher among females than males.

Studies on mice have indicated that female embryos are more susceptible to NTDs than males in some, but not all, genetic models.[3] For example, there is a higher rate of cranial NTDs in mice carrying mutations in *Trp53*,[6] *Pax3*, or *Nf1*[31] but not *Gldc*.[32] Sex differences in NTD susceptibility in *Trp53* null mice may result from the presence of two X chromosomes and may be independent of the Y chromosome.[6] Given the association between abnormal one-carbon metabolism or impaired methylation and NTDs, it has been hypothesized that the higher rate of cranial NTDs in females may be an epigenetic phenomenon. The demand for genomic DNA methylation is higher in females, which methylate most of the DNA in the large inactive X chromosome after every cell division, reducing the methylation available for other cellular needs.[2] In the *splotch* (Sp^{2H}; *Pax3* mutant) strain, female susceptibility to exencephaly is

For peer review only - http://bmjopen.bmj.com/site/about/guidelines.xhtml

exacerbated by dietary folate deficiency to a greater extent than in males.[33] While the etiology of human NTDs is much more complex, if such a scenario occurs in some human NTDs, then it might be predicted that an overall increase in population-level folate status may have a greater effect on NTDs in females than in males. This is consistent with our findings for anencephaly rates.

Genetic and environmental factors may also interact to determine murine sex ratios, with female embryos being more susceptible to loss. In the *splotch* strain, for example, arsenite treatment and advanced maternal age are both associated with higher rates of *in utero* death and NTDs.[34] Both factors result in elevated sex ratios (male:female) among litters, which suggests increased loss of female embryos by resorption. This effect is genotype-dependent such that a greater effect is noted as the number of *Sp* alleles increases. The sex ratio is lowered as the number of mutant Pax3 alleles increasess.

Our are from a population-based surveillance system, which covered all live births (28 or more complete gestational weeks), all stillbirths of at least 20 weeks' gestational age, and pregnancy terminations at any gestational age. This could give a more precise estimation of NTDs than hospital-based surveillance systems that include only live births, as the majority of fetuses with NTDs are terminated following prenatal diagnosis before 28 gestational weeks in China.[17]

Two main limitations of our study should be mentioned. First, we included data from only five counties, and the results may not be generalizable to the entire province or country. Second, we did not include the detailed subtypes of spina bifida, such as high-level and low-level lesions, which may show different patterns. A future study may be useful to address this question.

For peer review only

In conclusion, the prevalence of NTDs decreased in both sexes after the implementation of a massive FA supplementation program, but to a greater degree (both total NTDs and open NTDs) in females.

For peer review only

For peer review only - http://bmjopen.bmj.com/site/about/guidelines.xhtml

Competing Interests: None declared.

Funding: This work was supported in part by the National Key Research and Development Program, Ministry of Science and Technology, P.R. China (grant No. 2016YFC1000500), National Natural Science Foundation of China (No. 81373014, No. 81511130088 and No. 81202265) and Medical Research Council (United Kingdom, N003713). NG is supported by the MRC and Great Ormond Street Hospital Children's Charity.

For peer review only

References

1. Hay S. Sex differences in the incidence of certain congenital malformations: a review of the literature and some new data. *Teratology*. 1971; 4:277-286.

2. Juriloff DM, Harris MJ. Hypothesis: the female excess in cranial neural tube defects reflects an epigenetic drag of the inactivating X chromosome on the molecular mechanisms of neural fold elevation. *Birth Defects Res A Clin Mol Teratol*. 2012; 94:849-855.

3. Copp AJ, Stanier P, Greene ND. Neural tube defects: recent advances, unsolved questions, and controversies. *Lancet Neurol*. 2013; 12:799-810.

4. Seller MJ. Sex, neural tube defects, and multisite closure of the human neural tube. *Am J Med Genet*. 1995; 58:332-336.

5. Copp AJ, Brook FA. Does Lumbosacral Spina-Bifida Arise by Failure of Neural Folding or by Defective Canalization. *J Med Genet*. 1989; 26:160-166.

6. Chen X, Watkins R, Delot E, Reliene R, Schiestl RH, Burgoyne PS, et al. Sex difference in neural tube defects in p53-null mice is caused by differences in the complement of X not Y genes. *Dev Neurobiol*. 2008; 68:265-273.

7. Brook FA, Estibeiro JP, Copp AJ. Female predisposition to cranial neural tube defects is not because of a difference between the sexes in the rate of embryonic growth or development during neurulation. *J Med Genet*. 1994; 31:383-387.

8. Martinez de Villarreal LE, Arredondo P, Hernandez R, Villarreal JZ. Weekly administration of folic acid and epidemiology of neural tube defects. *Matern Child Health J*. 2006; 10:397-401.

9. Berry RJ, Li Z, Erickson JD, Li S, Moore CA, Wang H, et al. Prevention of neural-tube defects with folic acid in China. China-U.S. Collaborative Project for Neural Tube Defect Prevention. *N Engl J Med*. 1999; 341:1485-1490.

10. Smithells RW, Sheppard S, Schorah CJ, Seller MJ, Nevin NC, Harris R, et al. Apparent prevention of neural tube defects by periconceptional vitamin supplementation. 1981. *Int J Epidemiol*. 2011; 40:1146-1154.

11. MRC Vitamin Study Research Group. Prevention of neural tube defects: results of the Medical Research Council Vitamin Study. *Lancet*. 1991; 338:131-137.

12. Seller MJ. Multi-site neural tube closure in humans and maternal folate supplementation. *Am J Med Genet*. 1995; 58:222-224.

13. Honein MA, Paulozzi LJ, Mathews TJ, Erickson JD, Wong LY. Impact of folic acid fortification of the US food supply on the occurrence of neural tube defects. *JAMA*. 2001; 285:2981-2986.

14. Williams LJ, Mai CT, Edmonds LD, Shaw GM, Kirby RS, Hobbs CA, et al. Prevalence of spina bifida and anencephaly during the transition to mandatory folic acid fortification in the United States. *Teratology*. 2002; 66:33-39.

15. Xiao KZ, Zhang ZY, Su YM, Liu FQ, Yan ZZ, Jiang ZQ, et al. Central nervous system congenital malformations, especially neural tube defects in 29 provinces, metropolitan cities and autonomous regions of China: Chinese Birth Defects Monitoring Program. *Int J Epidemiol*. 1990; 19:978-982.

16. Li Z, Ren A, Zhang L, Ye R, Li S, Zheng J, et al. Extremely high prevalence of neural tube defects in a 4-county area in Shanxi Province, China. *Birth Defects Res A Clin Mol Teratol*. 2006; 76:237-240.

17. Li K, He XB, Zhang L, Li ZW, Ye RW, Liu JM, et al. [Impact of pregnancy terminations before 28 weeks of gestation on the overall prevalence of neural tube defects in two counties of Shanxi province]. *Zhonghua Liu Xing Bing Xue Za Zhi*. 2012; 33:509-512.

For peer review only - http://bmjopen.bmj.com/site/about/guidelines.xhtml

18. Ren AG. Prevention of neural tube defects with folic acid: The Chinese experience. *World J Clin Pediatr*. 2015; 4:41-44.

19. Liu J, Zhang L, Li Z, Jin L, Zhang Y, Ye R, et al. Prevalence and trend of neural tube defects in five counties in Shanxi province of Northern China, 2000 to 2014. *Birth Defects Res A Clin Mol Teratol*. 2016; 106:267-274.

20. Li S, Moore CA, Li Z, Berry RJ, Gindler J, Hong SX, et al. A population-based birth defects surveillance system in the People's Republic of China. *Paediatr Perinat Epidemiol*. 2003; 17:287-293.

21. Ren A, Zhang L, Hao L, Li Z, Tian YH, Li Z. Comparison of blood folate levels among pregnant Chinese women in areas with high and low prevalence of neural tube defects. *Public Health Nutr*. 2007; 10:762-768.

22. Liu J, Gao L, Zhang Y, Jin L, Li Z, Zhang L, et al. Plasma folate levels in early to mid pregnancy after a nation-wide folic acid supplementation program in areas with high and low prevalence of neural tube defects in China. *Birth Defects Res A Clin Mol Teratol*. 2015; 103:501-508.

23. O'Broin S, Kelleher B. Microbiological assay on microtitre plates of folate in serum and red cells. *J Clin Pathol*. 1992; 45:344-347.

24. Ashenfelter O, Card D. Using the Longitudinal Structure of Earnings to Estimate the Effect of Training Programs. *Review of Economics & Statistics*. 1985; 67:648-660.

25. Zhang L, Ren A, Li Z, Hao L, Tian Y, Li Z. Folate concentrations and folic acid supplementation among women in their first trimester of pregnancy in a rural area with a high prevalence of neural tube defects in Shanxi, China. *Birth Defects Res A Clin Mol Teratol*. 2006; 76:461-466.

26. Williams J, Mai CT, Mulinare J, Isenburg J, Flood TJ, Ethen M, et al. Updated estimates of neural tube defects prevented by mandatory folic Acid fortification - United States, 1995-2011. *MMWR Morb Mortal Wkly Rep*. 2015; 64:1-5.

27. Moore CA, Li S, Li Z, Hong SX, Gu HQ, Berry RJ, et al. Elevated rates of severe neural tube defects in a high-prevalence area in northern China. *Am J Med Genet*. 1997; 73:113-118.

28. Zeng Z, Zhu J. Low folic acid supplement intake rate among women in northern China with a high-prevalence of neural tube defects, 2008. *Prev Med*. 2010; 51:338-339.

29. Morris JK, Wald NJ. Prevalence of neural tube defect pregnancies in England and Wales from 1964 to 2004. *J Med Screen*. 2007; 14:55-59.

30. Bower C, D'Antoine H, Stanley FJ. Neural tube defects in Australia: trends in encephaloceles and other neural tube defects before and after promotion of folic acid supplementation and voluntary food fortification. *Birth Defects Res A Clin Mol Teratol*. 2009; 85:269-273.

31. Lakkis MM, Golden JA, O'Shea KS, Epstein JA. Neurofibromin deficiency in mice causes exencephaly and is a modifier for Splotch neural tube defects. *Dev Biol*. 1999; 212:80-92.

32. Pai YJ, Leung KY, Savery D, Hutchin T, Prunty H, Heales S, et al. Glycine decarboxylase deficiency causes neural tube defects and features of non-ketotic hyperglycinemia in mice. *Nat Commun*. 2015; 6:6388.

33. Burren KA, Savery D, Massa V, Kok RM, Scott JM, Blom HJ, et al. Gene-environment interactions in the causation of neural tube defects: folate deficiency increases susceptibility conferred by loss of Pax3 function. *Hum Mol Genet*. 2008; 17:3675-3685.

34. Martin LJ, Machado AF, Loza MA, Mao GE, Lee GS, Hovland DN, Jr., et al. Effect of arsenite, maternal age, and embryonic sex on spina bifida, exencephaly, and resorption rates in the splotch mouse. *Birth Defects Res A Clin Mol Teratol*. 2003; 67:231-239.

For peer review only - http://bmjopen.bmj.com/site/about/guidelines.xhtml

Figure legends

Figure 1. Folate concentration and NTD prevalence in Shanxi Province, China, in 2002–2012

Figure 2. Folate concentration separated by year and use of folic acid in Shanxi Province, China, in 2002–2012

For peer review only

For peer review only - http://bmjopen.bmj.com/site/about/guidelines.xhtml

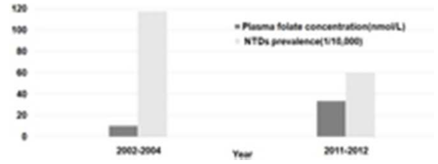

Figure 1. Folate concentration and NTD prevalence in Shanxi Province, China, in 2002–2012

8x3mm (600 x 600 DPI)

For peer review only

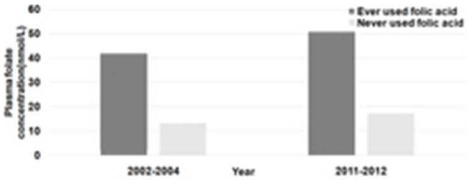

Figure 2. Folate concentration separated by year and use of folic acid in Shanxi Province, China, in 2002–2012

9x3mm (600 x 600 DPI)

For peer review only

Table 1. Prevalence Rates of NTDs (per 10,000 births) by Type and Sex in Shanxi Province, China, in 2003–2004 and 2011–2016

NTDs type	Male		Female		M:F rate ratio		
	N	Rate	N	Rate	RR	95% CI	
2003–2004(Pre-FA supplementation)							
**Open NTDs**	**145**	**110.3**	**168**	**138.9**	**0.79**	**0.77**	**0.81**
Anencephaly	65	49.4	94	77.7	0.78	0.76	0.80
Spina bifida	80	60.8	74	61.2	0.64	0.61	0.67
**Encephalocele**	**11**	**8.4**	**17**	**14.1**	**0.99**	**0.94**	**1.04**
**Total NTDs**	**156**	**118.6**	**185**	**152.9**	**0.60**	**0.45**	**0.80**
2011–2016(Post-FA supplementation)							
**Open NTDs**	**160**	**36.7**	**158**	**39.1**	**0.94**	**0.92**	**0.96**
Anencephaly	64	14.7	77	19.0	0.77	0.73	0.81
Spina bifida	96	22.0	81	20.0	1.10	1.05	1.15
**Encephalocele**	**17**	**3.9**	**21**	**5.2**	**0.75**	**0.61**	**0.92**
**Total NTDs**	**177**	**40.7**	**179**	**44.2**	**0.92**	**0.90**	**0.94**

For peer review only

Table 2. Differences in Changes in Prevalence Rates of NTDs (per 10,000 births) by Type and Sex in Shanxi Province, China, in 2003–2004 and 2011–2016

Change (from 2004 to 2016)	Male rate	Female rate	Female-Male DID Value	P
**Open NTDs**	73.5	99.8	26.3	0.001
Anencephaly	34.7	58.7	23.9	0.001
Spina bifida	38.8	41.1	2.4	0.765
**Encephalocele**	4.5	8.9	4.4	0.213
**Total NTDs**	78.0	108.7	30.7	0.007

Note: Rate of decrease in prevalence = Prevalence of group before folic acid supplementation – Prevalence of group after folic acid supplementation

For peer review only - http://bmjopen.bmj.com/site/about/guidelines.xhtml

BMJ Open

Sex differences in the prevalence of neural tube defects and preventive effects of folic acid (FA) supplementation among five counties in northern China: Results from a population-based birth defect surveillance program

Journal:	*BMJ Open*
Manuscript ID	bmjopen-2018-022565.R1
Article Type:	Research
Date Submitted by the Author:	12-May-2018
Complete List of Authors:	Liu, Jufen; Peking University, Institute of Reproductive and Child Health<br>Xie, Jing; Peking University, Institute of Reproductive and Child Health<br>Li, Zhiwen; Peking University, Institute of Reproductive and Child Health<br>Greene, ND; University College London Institute of Child Health, Birth Defects Research Centre<br>Ren, Aiguo; Peking University, Institute of Reproductive & Child Health
**Primary Subject Heading**:	Epidemiology
Secondary Subject Heading:	Nutrition and metabolism, Paediatrics, Public health, Reproductive medicine
Keywords:	EPIDEMIOLOGY, PUBLIC HEALTH, Neurogenetics < NEUROLOGY

SCHOLARONE™
Manuscripts

For peer review only

Title page

Title:

Sex differences in the prevalence of neural tube defects and preventive effects of folic acid (FA) supplementation among five counties in northern China: Results from a population-based birth defect surveillance program

Names of authors:

Jufen Liu, Ph.D. [1,2], Jing Xie, BD [1,2], Zhiwen Li, Ph.D. [1,2*], Nicholas D. E. Greene , Ph.D.[3], Aiguo Ren, Ph.D.[1,2*]

Affiliations of authors:

[1] Institute of Reproductive and Child Health / Key Laboratory of Reproductive Health, National Health Commission of the People's Republic of China, Peking University, Beijing 100191, P. R. China

[2] Department of Epidemiology and Biostatistics, School of Public Health, Peking University, Beijing 100191, P. R. China

[3] UCL Great Ormond Street Institute of Child Health, University College London, London WC1N 1EH, United Kingdom

[*] Zhiwen Li and Aiguo Ren contributed equally to this paper

[*]**Corresponding author:**

Zhiwen Li, 38 College Rd, Haidian District, Institute of Reproductive and Child Health, Peking University Health Science Center, Beijing 100191, China. Tel.: +86-10-82801140; Fax: +86-10-82801141. Email: lizw@bjmu.edu.cn

Author contributions:

Zhiwen Li designed research, Jufen Liu and Zhiwen Li conducted research, Jufen Liu analyzed the data and wrote the first draft of the manuscript, Jing Xie collected the data and analyzed the data, Aiguo Ren supervised data collection and revised the draft, Nicholas D. E. Greene revised the draft and provided critical suggestions on the study plan. All authors read, reviewed and approved the final manuscript.

For peer review only - http://bmjopen.bmj.com/site/about/guidelines.xhtml

Abstract

Objectives: Sex differences in prevalence of neural tube defects (NTDs) have previously been recognized; however the different susceptibility of males and females have not been examined in relation to the effects of folic acid (FA) supplementation. We hypothesized that FA may have a disproportionate effect that alters the sex-specific prevalence of NTDs.

Setting: Data from two time points, before (2003–2004) and after (2011–2016) the start of the supplementation program, were obtained from a population-based birth defect surveillance program among five counties in northern China. All pregnancies that reached 28 complete gestational weeks, including live births, stillbirths, and pregnancy terminations, and all NTDs regardless of gestational weeks were recorded.

Participants: A total of 25,249 and 82,258 births before and after the program were included respectively.

Primary and secondary outcome measures: The prevalence of NTDs by sex and subtype, Male:female sex ratios and their 95% confidence intervals were calculated.

Results: Overall, NTDs were less prevalent among males than among females (relative risk [RR], 0.92; 95% confidence interval [CI], 0.90–0.94), so was anencephaly (RR, 0.77; 95% CI, 0.73–0.81) and encephalocele (RR, 0.75; 95% CI, 0.61–0.92), while spina bifida showed a male predominance (RR, 1.10; 95% CI, 1.05–1.15). The overall prevalence of NTDs decreased by 78/10,000 in males and 108.7/10,000 in females from 2003–2004 to 2011–2016. There was a significant sex difference in the magnitude of reduction, being greater in females than males, particularly for anencephaly.

Conclusions: The prevalence of NTDs decreased in both sexes after the implementation of a massive FA supplementation program. While female predominance was observed in open NTDs and total NTDs, they also had a greater rate of decrease in NTDs after the supplementation program.

Key words: Sex differences, neural tube defects, folic acid supplementation

For peer review only - http://bmjopen.bmj.com/site/about/guidelines.xhtml

Strengths and limitations of this study

- Neural tube defects decreased significantly among both males and females after the implementation of a massive folic acid supplementation program in China.
- These decreases were significantly greater in females than in males.
- The reduction in NTD cases during the post-FA period inversely mirrors the increase in plasma folate concentrations among pregnant women.

For peer review only

For peer review only - http://bmjopen.bmj.com/site/about/guidelines.xhtml

INTRODUCTION

The prevalence of many types of birth defects appears to differ by sex, with the majority of them being more prevalent among males, [1 2] such as orofacial cleft (cleft lip with or without cleft palate), urinary system (hypospadias, hydronephrosis), and gastrointestinal tract (diaphragmatic hernia). However, some types of neural tube defects (NTDs) occur more frequently in females (e.g., isolated cases of cranial defects, such as anencephaly).[3] Females tend to have anencephaly more often than males, while craniorachischisis and spina bifida involving the higher spine may also show a preponderance among females. [4] By contrast, there tends to be a higher proportion of males than females with spina bifida affecting the lower spine. [4] There are thought to be multiple initiation sites in human neural tube closure, [5] as described in other mammalian embryos. Neural tube closure occurs during weeks 3–4 of human gestation and the sex of the embryo is differentially associated with a lack of closure of specific areas of the neural tube. [5]

Female predisposition to anencephaly is also observed among a number of mouse models, [6] but the mechanism underlying this has not been established. In mice, female embryos tend to be developmentally delayed compared to their male littermates at neurulation stages, leading to the hypothesis that the longer duration of cranial neural tube closure would lead to greater susceptibility to NTD-causing insult. However, going against this hypothesis, the delay in development may occur prior to neural tube closure, with female and male embryos taking the same time to complete cranial neurulation. [7] Other suggested mechanisms include sex differences in genetic risk, epigenetic factors, a differential rate of fetal loss, and susceptibility to environmental influences. [8]

Among environmental factors, periconceptional supplementation with folic acid (FA) has been shown to dramatically reduce the risk for NTDs in offspring [9] and conversely, suboptimal maternal folate status is a risk factor for NTDs. [10] Although a large

For peer review only - http://bmjopen.bmj.com/site/about/guidelines.xhtml

number of studies have investigated the preventive effects of FA on NTDs, the influence of maternal FA supplementation on sex-associated risk for NTDs and their subtypes is unclear. Some studies have lacked data on the type of NTDs or sex, [11] while others have not found significant differences among subtypes of NTD [12]. In these studies the apparent protective effects of FA does not seem to act preferentially in one region of the neural tube and there appears to be a general reduction in the occurrence of human NTDs.[13] In a study in Mexico in which weekly administration of 5 mg FA was associated with a 50% reduction in the incidence of NTDs, the main effect was on spina bifida, with a higher reduction in female cases.[8] In several countries folic acid fortification (FAF) of food has been associated with a decreased frequency of spina bifida and anencephaly on a population level. [14 15] A recent study from South America showed that prevalence of NTDs, particularly anencephaly and cervico-thoracic spina bifida, showed a greater reduction rate in females than in males after FAF resulting in a change of the sex ratio of infants with NTDs [16].

In 2009, the Ministry of Health of China initiated a nationwide FA supplementation program for the purpose of further decreasing the occurrence of NTDs in the country. [17] Women with a household registration and planning to become pregnant are eligible to obtain FA supplements free of charge through the maternal health care system. In a recent study, we showed that birth prevalence of NTDs in the five counties of Shanxi Province in northern China dropped dramatically, from 118.9/10,000 in 2000 to 31.5/10,000 in 2014. [18] In the present study, we examined the trends in NTD prevalence by sex in five counties of Shanxi Province, China, from 2003 to 2016, encompassing the introduction of a massive FA supplementation campaign. Characterization of the sex distribution of NTDs may be helpful to provide personalized prevention recommendations for NTDs, and to better understand NTD development in association with FA supplementation.

MATERIALS AND METHODS

Birth defect surveillance

Five counties in Shanxi Province (Pingding, Xiyang, Taigu, Zezhou, and Shouyang)

For peer review only - http://bmjopen.bmj.com/site/about/guidelines.xhtml

were included in a population-based birth defects surveillance system in the current study. The surveillance system was similar to a previously reported system, created to meet the primary objective of monitoring the prevalence of major structural birth defects including NTDs.[19] A national FA supplementation program was initiated in China in 2009; therefore, in the current study we collected data at two time points: before (2003–2004) and after (2011–2016) the start of the supplementation program. All pregnant women who resided in the study area for more than 1 year during the period were monitored. All live births (28 or more complete gestational weeks), all stillbirths of at least 20 weeks' gestational age, and pregnancy terminations at any gestational age following the prenatal diagnosis of NTDs were included. Photographs of every suspected case were obtained. NTD diagnosis was conducted by local specialists in maternal-fetal medicine, and photographs were sent to a pediatrician at Peking University for confirmation.

Two cross-sectional survey data

In addition to the NTD prevalence data, we also obtained cross-sectional survey data on the use of FA and blood folate concentration among pregnant women in the two periods, to provide external evidence to test the correlation between folate status and NTDs. Those two surveys took place in the same area, one before the FA program started (2003–2004) [20] and the other after it started (2011–2012). [21] Detailed descriptions of the two studies have already been published.[21 22] Briefly, A trained interviewer conducted a face-to-face interview with a structured questionnaire after obtaining consent from the women who was in their late first trimester or early second trimester of pregnancy. Information on folic acid supplementation, timing of supplementation, frequency of supplementation, and days of supplementation was collected. A 5-ml non fasting blood sample was collected and drawn into K3EDTA-containing Vacutainer tubes (Becton Dickinson) at the time of recruitment. Plasma was separated and temporarily stored at -20℃ in local hospitals, and subsequently transferred to our laboratory and stored at -80℃ until assessment. Plasma levels of folate were determined using a microbiological assay.[23 21] Intra and

For peer review only - http://bmjopen.bmj.com/site/about/guidelines.xhtml

inter-assay coefficients of variation were ≤10%. The study protocol was reviewed and approved by the Institutional Review Board of Peking University.

Statistical analysis

The prevalence of NTDs by infant sex was analyzed separately for open NTDs (anencephaly, spina bifida) [24] and encephaloecele. The denominator was the total number of all births and pregnancy that reached 28 or more complete gestational weeks by sex; the numerator was the number of NTDs cases regardless of gestational age by sex. The prevalence of NTDs by sex and subtype were compared using Chi-square tests. A difference in differences (DID) model was applied to compare sex differences between the time periods. [25] A two-tailed $p \leq 0.05$ was considered to indicate statistically significant differences. All statistical analyses were performed using the SPSS package version 20.0 (SPSS Inc., Chicago, USA).

Patient and Public Involvement

Our main analysis was based on the birth defects surveillance system; the information was anonymized. Patients and or public were not involved.

RESULTS

Prevalence Before FA Supplementation Program

The prevalence of NTDs by subtype and sex are shown in Table 1. Before the program, a total of 25,249 births and 341 cases of NTDs (156 males, 185 females) were recorded in the system. The overall prevalence of NTDs for both sexes was 135.1/10,000, comprising 118.6/10,000 among males and 152.9/10,000 among females. All subtypes except spina bifida were more prevalent among females with a male-to-female RR of 0.60 (95% CI, 0.45–0.80) and 0.79 (95% CI, 0.77–0.81) for total NTDs and open NTDs, respectively (Table 1).

For peer review only - http://bmjopen.bmj.com/site/about/guidelines.xhtml

Table 1. Prevalence Rates of NTDs (per 10,000 births) by Type and Sex in Shanxi Province, China, in 2003–2004 and 2011–2016

NTDs type	Male			Female			M:F rate ratio		
	Births	Cases	Rate	Births	Cases	Rate	RR	95% CI	
2003–2004 (Pre-FA supplementation)									
	13 150			12 099					
Open NTDs		145	110.3		168	138.9	0.79	0.77	0.81
Anencephaly		65	49.4		94	77.7	0.64	0.61	0.67
Spina bifida		80	60.8		74	61.2	0.99	0.94	1.04
Encephalocele		11	8.4		17	14.1	0.60	0.45	0.80
Total NTDs		156	118.6		185	152.9	0.78	0.76	0.80
2011–2016 (Post-FA supplementation)									
	43 538			40 458					
Open NTDs		160	36.7		158	39.1	0.94	0.92	0.96
Anencephaly		64	14.7		77	19.0	0.77	0.73	0.81
Spina bifida		96	22.0		81	20.0	1.10	1.05	1.15
Encephalocele		17	3.9		21	5.2	0.75	0.61	0.92
Total NTDs		177	40.7		179	44.2	0.92	0.90	0.94

For peer review only

Prevalence During FA Supplementation Program

After the initiation of the program, a total of 82,258 births and 356 cases of NTDs (177 males, 179 females) were recorded in the system. The overall prevalence for both sexes was 43.3/10,000, being 40.7/10,000 among males and 44.2/10,000 among females. Hence, in sum, NTDs were less prevalent among males than females (RR, 0.92; 95% CI, 0.90–0.94), so was anencephaly (RR, 0.77; 95% CI, 0.73–0.81) and encephalocele (RR, 0.75; 95% CI, 0.61–0.92), while spina bifida showed male predominance (RR, 1.10; 95% CI, 1.05–1.15) (Table 1).

Differences in Changes in Prevalence Rates of NTDs

After the supplementation period, the prevalence of all types of NTDs was lower in both male and female fetuses. However, there was a significant sex difference in the magnitude of reduction, being more pronounced among females, especially for open NTDs including anencephaly ($p<0.01$). The overall prevalence of NTDs decreased by 78/10,000 in males and 108.7/10,000 in females in absolute terms; anencephaly decreased by 34.7% in males and 58.7% in females in relative term (Table 2).

For peer review only - http://bmjopen.bmj.com/site/about/guidelines.xhtml

Table 2. Differences in Changes in Prevalence Rates of NTDs (per 10,000 births) by Type and Sex in Shanxi Province, China, in 2003–2004 and 2011–2016

Types	Male rate		Female rate		Female-Male DID Value	P
	Absolute	Relative	Absolute	Relative		
**Open NTDs**	73.5	66.7	99.8	71.9	26.3	0.001
Anencephaly	34.7	70.3	58.7	75.5	23.9	0.001
Spina bifida	38.8	63.8	41.1	67.3	2.4	0.765
**Encephalocele**	4.5	53.3	8.9	63.1	4.4	0.213
**Total NTDs**	78.0	65.7	108.7	71.1	30.7	0.007

Note:

1. Absolute rate of decrease in prevalence = Prevalence of group before folic acid supplementation – Prevalence of group after folic acid supplementation
2. Relative rate of decrease in prevalence = Absolute rate of decrease in prevalence/Prevalence of group before folic acid supplementation

DISCUSSION

This quasi-experimental population-based study examined whether introduction of population-wide FA supplementation was associated with changes in the sex bias among NTDs in northern China, as a means to understand whether the preventive effects of FA supplementation differ by sex. We found that, after the introduction of the national FA supplementation campaign, the prevalence of total NTDs and open NTDs (including anencephaly, spina bifida) decreased among both sexes. However, the magnitude of decline was significantly greater among females than males.

The prevalence of NTDs in Shanxi Province in northern China is historically high, being 105.5 per 10,000 births in the late 1980s [26] and 138 per 10,000 births in 2003.[27] However, differences between the sexes among this population have rarely been examined. One previous study reported a female predominance among anencephaly cases (male-to-female relative risk [RR], 0.49; 95% confidence interval [CI], 0.30–0.79) but not among spina bifida (RR, 0.90; 95% CI, 0.55–1.45) or encephalocele (RR, 1.03; 95% CI, 0.40–2.69) cases in 2006; [27] however, that study was conducted before the national FA supplementation program and how the NTD prevalence has changed in each sex in association with the program remains unknown.

For peer review only - http://bmjopen.bmj.com/site/about/guidelines.xhtml

Since 2009, the nationwide FA supplementation program has provided FA supplements, free of charge, to all women who have a rural registration and who plan to become pregnant. As we previously reported, the prevalence of NTDs was dramatically lower in 2012–2014 than in 2009 in Shanxi Province. This decrease may be partially attributable to the program; however, this decreasing trend has not continued since 2014. In fact, the prevalence in the same area was higher in 2016 (31.5 per 10,000 births) than in 2014 (31.5 per 10,000 births). Moreover, the prevalence of NTDs in this area remains high when compared to the rates in the USA, [15] Canada, [28] Germany, [29] England, Wales, [30] and Western Australia.[31] The compliance of women taking the folic acid pills may be attributable to the high prevalence. A previous study showed the percentage of folic acid supplementation before the last menstrual period increased only from 30% to 43% and the supplementation adherence of ⩾8 days/10 days showed a decrease. [32] Other reasons may need further study.

The prevalence of NTDs was lower among both males and females after the program. FA supplementation was successful in that the proportion of people taking FA supplements increased from 9.2% in 2002–2004 [33] to 66.3% in 2011–2012 [21]. In Shanxi Province, plasma folate concentrations were significantly higher after the program, and NTD prevalence was significantly lower. However, the effect was not the same for males and females, being greater among females than males. For open NTDs, the decrease was 26.3/10,000 higher among females than males; for total NTDs (including encephalocele), the decrease was 30.7/10,000 higher among females than males.

Comparison of population data on blood folate concentration among pregnant women and NTD prevalence data indicated a clear trend: plasma folate concentration increased from 10.4 nmol/L in 2003–2004 [33] to 33.4 nmol/L in 2011–2012[22] while the prevalence of NTDs decreased from 117.8/10,000 births to 60.3/10,000 births, respectively[19]. Groups who reported taking FA supplements had significantly higher folate concentrations (median 41.9 nmol/L) than those who did not take FA (13.2

For peer review only - http://bmjopen.bmj.com/site/about/guidelines.xhtml

nmol/L) during the two time periods. In contrast, these values were 50.7 nmol/L and 17.3 nmol/L, respectively, in 2011–2012.

Studies on mice have indicated that female embryos are more susceptible to NTDs than males in some, but not all, genetic models. [34] For example, there is a higher rate of cranial NTDs in mice carrying mutations in *Trp53*, [6] *Pax3*, or *Nf1* [35] but not *Gldc*.[36] Sex differences in NTD susceptibility in *Trp53* null mice may result from the presence of two X chromosomes and independent of the Y chromosome.[6] Given the association between abnormal one-carbon metabolism or impaired methylation and NTDs, it has been hypothesized that the higher rate of cranial NTDs in females may be an epigenetic phenomenon. The demand for genomic DNA methylation is higher in females, which methylate most of the DNA in the large inactive X chromosome after every cell division. It is speculated that this may reduce the methylation capacity available for other key cellular needs.[3] In the *splotch* (Sp^{2H}; *Pax3* mutant) strain, female susceptibility to exencephaly is exacerbated by dietary folate deficiency to a greater extent than in males. [37] While the etiology of human NTDs is more complex than in single-gene models, if such a scenario occurs in some human NTDs, then it might be predicted that an overall increase in population-level folate status may have a greater effect on NTDs in females than in males. This is consistent with our findings for anencephaly rates.

Genetic and environmental factors may also interact to determine murine sex ratios, with female embryos being more susceptible to lethality. In the *splotch* strain, for example, arsenite treatment and advanced maternal age are both associated with higher rates of *in utero* death and NTDs. [38] Both factors result in elevated sex ratios (male:female) among litters, which suggests increased loss of female embryos by resorption. This effect is genotype-dependent such that a greater effect, with lower sex ratio, is noted as the number of *Sp* (*Pax3* mutant) alleles increases. Study of human NTDs also indicated an overall excess of affected females. [39] To fully understand the

For peer review only - http://bmjopen.bmj.com/site/about/guidelines.xhtml

genetic influences of NTDs, future studies are needed to investigate the importance of epigenetic factors and imprinting effects, methylation status in altering NTD risk.

Our data are from a population-based surveillance system, which covered all live births (28 or more complete gestational weeks), and NTD cases from pregnancies of any gestational age. This could give a more precise estimation of NTD rate than hospital-based surveillance systems that include only NTD cases in pregnancies of 28 gestational weeks or greater in China. [40]

Three main limitations of our study should be mentioned. First, we included data from only five counties, and the results may not be generalizable to the entire province or country. Second, we did not include the detailed subtypes of spina bifida, such as high-level and low-level lesions, which may show different patterns. A future study may be useful to address this question. Third, as an ecological design in which some confounding factors might affect our results, the massive FA supplementation program was the only major factor that may affect the population rate of NTDs in the study. Improvement in living standards and prenatal diagnosis may also impact the rate of NTDs, but these potential factors is not predicted to have a differential effect on males and females.

In conclusion, the prevalence of NTDs decreased in both sexes after the implementation of a massive FA supplementation program, but to a greater degree (both total NTDs and open NTDs) in females.

Statement on ethical approval and data sharing:

Our study protocol was reviewed and approved by the Institutional Review Board of Peking University. Dr. Zhiwen Li took full responsibility to the data and data could be shared upon requirement.

For peer review only - http://bmjopen.bmj.com/site/about/guidelines.xhtml

Competing Interests: None declared.

Funding: This work was supported in part by the National Key Research and Development Program, Ministry of Science and Technology, P.R. China (grant No. 2016YFC1000500), National Natural Science Foundation of China (No. 81373014, No. 81511130088 and No. 81202265) and Medical Research Council (United Kingdom, N003713). NG is supported by the MRC and Great Ormond Street Hospital Children's Charity.

For peer review only

References

1. Hay S. Sex differences in the incidence of certain congenital malformations: a review of the literature and some new data. *Teratology* 1971;4(3):277-86. doi: 10.1002/tera.1420040303
2. Michalski AM, Richardson SD, Browne ML, et al. Sex Ratios Among Infants with Birth Defects, National Birth Defects Prevention Study, 1997-2009. *Am J Med Genet A* 2015;167(5):1071-81. doi: 10.1002/ajmg.a.36865
3. Juriloff DM, Harris MJ. Hypothesis: the female excess in cranial neural tube defects reflects an epigenetic drag of the inactivating X chromosome on the molecular mechanisms of neural fold elevation. *Birth defects research Part A, Clinical and molecular teratology* 2012;94(10):849-55. doi: 10.1002/bdra.23036
4. Tennant PW, Samarasekera SD, Pless-Mulloli T, et al. Sex differences in the prevalence of congenital anomalies: a population-based study. *Birth defects research Part A, Clinical and molecular teratology* 2011;91(10):894-901. doi: 10.1002/bdra.22846 [published Online First: 2011/10/12]
5. Seller MJ. Sex, neural tube defects, and multisite closure of the human neural tube. *American journal of medical genetics* 1995;58(4):332-6. doi: 10.1002/ajmg.1320580406 [published Online First: 1995/09/25]
6. Chen X, Watkins R, Delot E, et al. Sex difference in neural tube defects in p53-null mice is caused by differences in the complement of X not Y genes. *Developmental neurobiology* 2008;68(2):265-73. doi: 10.1002/dneu.20581 [published Online First: 2007/11/16]
7. Brook FA, Estibeiro JP, Copp AJ. Female predisposition to cranial neural tube defects is not because of a difference between the sexes in the rate of embryonic growth or development during neurulation. *Journal of medical genetics* 1994;31(5):383-7. [published Online First: 1994/05/01]
8. Martinez de Villarreal LE, Arredondo P, Hernandez R, et al. Weekly administration of folic acid and epidemiology of neural tube defects. *Matern Child Health J* 2006;10(5):397-401. doi: 10.1007/s10995-006-0082-2
9. Berry RJ, Li Z, Erickson JD, et al. Prevention of neural-tube defects with folic acid in China. China-U.S. Collaborative Project for Neural Tube Defect Prevention. *N Engl J Med* 1999;341(20):1485-90. doi: 10.1056/NEJM199911113412001
10. Crider KS, Devine O, Hao L, et al. Population red blood cell folate concentrations for prevention of neural tube defects: Bayesian model. *BMJ* 2014;349:g4554. doi: 10.1136/bmj.g4554
11. Smithells RW, Sheppard S, Schorah CJ, et al. Apparent prevention of neural tube defects by periconceptional vitamin supplementation. 1981. *Int J Epidemiol* 2011;40(5):1146-54. doi: 10.1093/ije/dyr143
12. MRC Vitamin Study Research Group. Prevention of neural tube defects: results of the Medical Research Council Vitamin Study. . *Lancet* 1991;338(8760):131-7.
13. Seller MJ. Multi-site neural tube closure in humans and maternal folate supplementation. *American journal of medical genetics* 1995;58(3):222-4. doi: 10.1002/ajmg.1320580305
14. Honein MA, Paulozzi LJ, Mathews TJ, et al. Impact of folic acid fortification of the US food supply on the occurrence of neural tube defects. *JAMA* 2001;285(23):2981-6.
15. Williams J, Mai CT, Mulinare J, et al. Updated estimates of neural tube defects prevented by mandatory folic Acid fortification - United States, 1995-2011. *MMWR Morb Mortal Wkly Rep*

For peer review only - http://bmjopen.bmj.com/site/about/guidelines.xhtml

2015;64(1):1-5.

16. Poletta FA, Rittler M, Saleme C, et al. Neural tube defects: Sex ratio changes after fortification with folic acid. *PLoS One* 2018;13(3):e0193127. doi: 10.1371/journal.pone.0193127
17. Ren AG. Prevention of neural tube defects with folic acid: The Chinese experience. *World J Clin Pediatr* 2015;4(3):41-4. doi: 10.5409/wjcp.v4.i3.41
18. Liu J, Zhang L, Li Z, et al. Prevalence and trend of neural tube defects in five counties in Shanxi province of Northern China, 2000 to 2014. *Birth defects research Part A, Clinical and molecular teratology* 2016;106(4):267-74. doi: 10.1002/bdra.23486
19. Li S, Moore CA, Li Z, et al. A population-based birth defects surveillance system in the People's Republic of China. *Paediatric and perinatal epidemiology* 2003;17(3):287-93.
20. Ren A, Zhang L, Hao L, et al. Comparison of blood folate levels among pregnant Chinese women in areas with high and low prevalence of neural tube defects. *Public health nutrition* 2007;10(8):762-68. doi: 10.1017/S1368980007246786
21. Liu J, Gao L, Zhang Y, et al. Plasma folate levels in early to mid pregnancy after a nation-wide folic acid supplementation program in areas with high and low prevalence of neural tube defects in China. *Birth Defects Res A Clin Mol Teratol* 2015;103(6):501-8. doi: 10.1002/bdra.23368
22. O'Broin S, Kelleher B. Microbiological assay on microtitre plates of folate in serum and red cells. *J Clin Pathol* 1992;45(4):344-7.
23. Ren AG, Zhang L, Li ZW, et al. Awareness and use of folic acid, and blood folate concentrations among pregnant women in northern China - An area with a high prevalence of neural tube defects. *Reprod Toxicol* 2006;22(3):431-36. doi: 10.1016/j.reprotox.2006.02.003
24. McComb JG. A practical clinical classification of spinal neural tube defects. *Child's nervous system : ChNS : official journal of the International Society for Pediatric Neurosurgery* 2015;31(10):1641-57. doi: 10.1007/s00381-015-2845-9 [published Online First: 2015/09/10]
25. Ashenfelter O, Card D. Using the Longitudinal Structure of Earnings to Estimate the Effect of Training-Programs. *Rev Econ Stat* 1985;67(4):648-60. doi: Doi 10.2307/1924810
26. Xiao KZ, Zhang ZY, Su YM, et al. Central-Nervous-System Congenital-Malformations, Especially Neural-Tube Defects in 29 Provinces, Metropolitan Cities and Autonomous Regions of China - Chinese-Birth-Defects-Monitoring-Program. *International Journal of Epidemiology* 1990;19(4):978-82.
27. Li Z, Ren A, Zhang L, et al. Extremely high prevalence of neural tube defects in a 4-county area in Shanxi Province, China. *Birth defects research Part A, Clinical and molecular teratology* 2006;76(4):237-40. doi: 10.1002/bdra.20248
28. De Wals P, Tairou F, Van Allen MI, et al. Reduction in neural-tube defects after folic acid fortification in Canada. *N Engl J Med* 2007;357(2):135-42. doi: 10.1056/NEJMoa067103
29. Nasri K, Ben Fradj MK, Hamdi T, et al. Epidemiology of neural tube defect subtypes in Tunisia, 1991-2011. *Pathol Res Pract* 2014;210(12):944-52. doi: 10.1016/j.prp.2014.06.027
30. Morris JK, Wald NJ. Prevalence of neural tube defect pregnancies in England and Wales from 1964 to 2004. *J Med Screen* 2007;14(2):55-9. doi: 10.1258/096914107781261945
31. Bower C, D'Antoine H, Stanley FJ. Neural Tube Defects in Australia: Trends in Encephaloceles and Other Neural Tube Defects Before and After Promotion of Folic Acid Supplementation and Voluntary Food Fortification. *Birth Defects Res A* 2009;85(4):269-73. doi: 10.1002/bdra.20536
32. Liu JF, Jin L, Meng QQ, et al. Changes in folic acid supplementation behaviour among women of reproductive age after the implementation of a massive supplementation programme in

For peer review only - http://bmjopen.bmj.com/site/about/guidelines.xhtml

China. *Public health nutrition* 2015;18(4):582-88. doi: 10.1017/S1368980014000950

33. Zhang L, Ren A, Li Z, et al. Folate concentrations and folic acid supplementation among women in their first trimester of pregnancy in a rural area with a high prevalence of neural tube defects in Shanxi, China. *Birth Defects Res A Clin Mol Teratol* 2006;76(6):461-6. doi: 10.1002/bdra.20271
34. Copp AJ, Stanier P, Greene ND. Neural tube defects: recent advances, unsolved questions, and controversies. *The Lancet Neurology* 2013;12(8):799-810. doi: 10.1016/s1474-4422(13)70110-8 [published Online First: 2013/06/25]
35. Lakkis MM, Golden JA, O'Shea KS, et al. Neurofibromin deficiency in mice causes exencephaly and is a modifier for Splotch neural tube defects. *Dev Biol* 1999;212(1):80-92. doi: 10.1006/dbio.1999.9327
36. Pai YJ, Leung KY, Savery D, et al. Glycine decarboxylase deficiency causes neural tube defects and features of non-ketotic hyperglycinemia in mice. *Nat Commun* 2015;6:6388. doi: 10.1038/ncomms7388
37. Burren KA, Savery D, Massa V, et al. Gene-environment interactions in the causation of neural tube defects: folate deficiency increases susceptibility conferred by loss of Pax3 function. *Hum Mol Genet* 2008;17(23):3675-85. doi: 10.1093/hmg/ddn262
38. Martin LJ, Machado AF, Loza MA, et al. Effect of arsenite, maternal age, and embryonic sex on spina bifida, exencephaly, and resorption rates in the splotch mouse. *Birth defects research Part A, Clinical and molecular teratology* 2003;67(4):231-9. doi: 10.1002/bdra.10006 [published Online First: 2003/07/12]
39. Deak KL, Siegel DG, George TM, et al. Further evidence for a maternal genetic effect and a sex-influenced effect contributing to risk for human neural tube defects. *Birth defects research Part A, Clinical and molecular teratology* 2008;82(10):662-9. doi: 10.1002/bdra.20511 [published Online First: 2008/10/22]
40. Li K, He XB, Zhang L, et al. [Impact of pregnancy terminations before 28 weeks of gestation on the overall prevalence of neural tube defects in two counties of Shanxi province]. *Zhonghua liu xing bing xue za zhi = Zhonghua liuxingbingxue zazhi* 2012;33(5):509-12. [published Online First: 2012/08/14]

For peer review only

For peer review only - http://bmjopen.bmj.com/site/about/guidelines.xhtml

STROBE Statement—Checklist of items that should be included in reports of ***cross-sectional studies***

Pages		Item No	Recommendation
1-2	**Title and abstract**	1	(*a*) Indicate the study's design with a commonly used term in the title or the abstract
			(*b*) Provide in the abstract an informative and balanced summary of what was done and what was found
	**Introduction**		
4-5	Background/rationale	2	Explain the scientific background and rationale for the investigation being reported
5	Objectives	3	State specific objectives, including any prespecified hypotheses
	**Methods**		
6	Study design	4	Present key elements of study design early in the paper
5-6	Setting	5	Describe the setting, locations, and relevant dates, including periods of recruitment, exposure, follow-up, and data collection
6	Participants	6	(*a*) Give the eligibility criteria, and the sources and methods of selection of participants
6	Variables	7	Clearly define all outcomes, exposures, predictors, potential confounders, and effect modifiers. Give diagnostic criteria, if applicable
6	Data sources/ measurement	8*	For each variable of interest, give sources of data and details of methods of assessment (measurement). Describe comparability of assessment methods if there is more than one group
6	Bias	9	Describe any efforts to address potential sources of bias
6	Study size	10	Explain how the study size was arrived at
6	Quantitative variables	11	Explain how quantitative variables were handled in the analyses. If applicable, describe which groupings were chosen and why
7	Statistical methods	12	(*a*) Describe all statistical methods, including those used to control for confounding
7			(*b*) Describe any methods used to examine subgroups and interactions
Not applicable			(*c*) Explain how missing data were addressed
Not applicable			(*d*) If applicable, describe analytical methods taking account of sampling strategy
Not applicable			(*e*) Describe any sensitivity analyses
	**Results**		
7-8	Participants	13*	(a) Report numbers of individuals at each stage of study—eg numbers potentially eligible, examined for eligibility, confirmed eligible, included in the study, completing follow-up, and analysed
Not applicable			(b) Give reasons for non-participation at each stage
Not applicable			(c) Consider use of a flow diagram
7-8	Descriptive data	14*	(a) Give characteristics of study participants (eg demographic, clinical, social) and information on exposures and potential confounders
Not			(b) Indicate number of participants with missing data for each variable

For peer review only

applicable			of interest
7-9	Outcome data	15*	Report numbers of outcome events or summary measures
7-9	Main results	16	(*a*) Give unadjusted estimates and, if applicable, confounder-adjusted estimates and their precision (eg, 95% confidence interval). Make clear which confounders were adjusted for and why they were included
7-9			(*b*) Report category boundaries when continuous variables were categorized
7-9			(*c*) If relevant, consider translating estimates of relative risk into absolute risk for a meaningful time period
8-9	Other analyses	17	Report other analyses done—eg analyses of subgroups and interactions, and sensitivity analyses
	**Discussion**		
9	Key results	18	Summarise key results with reference to study objectives
12	Limitations	19	Discuss limitations of the study, taking into account sources of potential bias or imprecision. Discuss both direction and magnitude of any potential bias
9-12	Interpretation	20	Give a cautious overall interpretation of results considering objectives, limitations, multiplicity of analyses, results from similar studies, and other relevant evidence
11-12	Generalisability	21	Discuss the generalisability (external validity) of the study results
	**Other information**		
13	Funding	22	Give the source of funding and the role of the funders for the present study and, if applicable, for the original study on which the present article is based

*Give information separately for exposed and unexposed groups.

Note: An Explanation and Elaboration article discusses each checklist item and gives methodological background and published examples of transparent reporting. The STROBE checklist is best used in conjunction with this article (freely available on the Web sites of PLoS Medicine at http://www.plosmedicine.org/, Annals of Internal Medicine at http://www.annals.org/, and Epidemiology at http://www.epidem.com/). Information on the STROBE Initiative is available at www.strobe-statement.org.

For peer review only

BMJ Open

Sex differences in the prevalence of neural tube defects and preventive effects of folic acid (FA) supplementation among five counties in northern China: Results from a population-based birth defect surveillance program

Journal:	*BMJ Open*
Manuscript ID	bmjopen-2018-022565.R2
Article Type:	Research
Date Submitted by the Author:	07-Aug-2018
Complete List of Authors:	Liu, Jufen; Peking University, Institute of Reproductive and Child Health<br>Xie, Jing; Peking University, Institute of Reproductive and Child Health<br>Li, Zhiwen; Peking University, Institute of Reproductive and Child Health<br>Greene, ND; University College London Institute of Child Health, Birth Defects Research Centre<br>Ren, Aiguo; Peking University, Institute of Reproductive & Child Health
**Primary Subject Heading**:	Epidemiology
Secondary Subject Heading:	Nutrition and metabolism, Paediatrics, Public health, Reproductive medicine
Keywords:	EPIDEMIOLOGY, PUBLIC HEALTH, Neurogenetics < NEUROLOGY

SCHOLARONE™
Manuscripts

For peer review only

Title page

Title:

Sex differences in the prevalence of neural tube defects and preventive effects of folic acid (FA) supplementation among five counties in northern China: Results from a population-based birth defect surveillance program

Names of authors:

Jufen Liu, Ph.D. [1,2], Jing Xie, BD [1,2], Zhiwen Li, Ph.D.[1,2*], Nicholas D. E. Greene , Ph.D.[3],Aiguo Ren, Ph.D.[1,2*]

Affiliations of authors:

[1] Institute of Reproductive and Child Health / Key Laboratory of Reproductive Health, National Health Commission of the People's Republic of China, Peking University, Beijing 100191, P. R. China

[2] Department of Epidemiology and Biostatistics, School of Public Health, Peking University, Beijing 100191, P. R. China

[3] UCL Great Ormond Street Institute of Child Health, University College London, London WC1N 1EH, United Kingdom

[*] Zhiwen Li and Aiguo Ren contributed equally to this paper

[*]**Corresponding author:**

Zhiwen Li, 38 College Rd, Haidian District, Institute of Reproductive and Child Health, Peking University Health Science Center, Beijing 100191, China. Tel.: +86-10-82801140; Fax: +86-10-82801141. Email: lizw@bjmu.edu.cn

Running head: Sex differences in NTDs in northern China

Author contributions:

Zhiwen Li designed research, Jufen Liu and Zhiwen Li conducted research, Jufen Liu analyzed the data and wrote the first draft of the manuscript, Jing Xie collected the data and analyzed the data, Aiguo Ren supervised data collection and revised the draft, Nicholas D. E. Greene revised the draft and provided critical suggestions on the study

For peer review only

plan. All authors read, reviewed and approved the final manuscript.

Word counts: 2940

For peer review only

For peer review only - http://bmjopen.bmj.com/site/about/guidelines.xhtml

Abstract

Objectives: Sex differences in prevalence of neural tube defects (NTDs) have previously been recognized; however the different susceptibility of males and females have not been examined in relation to the effects of folic acid (FA) supplementation. We hypothesized that FA may have a disproportionate effect that alters the sex-specific prevalence of NTDs.

Setting: Data from two time points, before (2003–2004) and after (2011–2016) the start of the supplementation program, were obtained from a population-based birth defect surveillance program among five counties in northern China. All live births (28 or more complete gestational weeks), all stillbirths of at least 20 weeks' gestational age, and pregnancy terminations at any gestational age following the prenatal diagnosis of NTDs were included.

Participants: A total of 25,249 and 82,258 births before and after the program were included respectively.

Primary and secondary outcome measures: The prevalence of NTDs by sex and subtype, Male:female sex ratios and their 95% confidence intervals were calculated.

Results: Overall, NTDs were less prevalent among males than among females (relative risk [RR], 0.92; 95% confidence interval [CI], 0.90–0.94), so was anencephaly (RR, 0.77; 95% CI, 0.73–0.81) and encephalocele (RR, 0.75; 95% CI, 0.61–0.92), while spina bifida showed a male predominance (RR, 1.10; 95% CI, 1.05–1.15). The overall prevalence of NTDs decreased by 78/10,000 in males and 108.7/10,000 in females from 2003–2004 to 2011–2016. There was a significant sex difference in the magnitude of reduction, being greater in females than males, particularly for anencephaly.

Conclusions: The prevalence of NTDs decreased in both sexes after the implementation of a massive FA supplementation program. While female predominance was observed in open NTDs and total NTDs, they also had a greater rate of decrease in NTDs after the supplementation program.

Keywords: Sex differences, neural tube defects, folic acid supplementation

For peer review only - http://bmjopen.bmj.com/site/about/guidelines.xhtml

Strengths and limitations of this study

- Our data are from a population-based surveillance system, which covered all live births (28 or more complete gestational weeks), and NTD cases from pregnancies of any gestational age.
- Neural tube defects decreased among both males and females after the implementation of a massive folic acid supplementation program in China, while these decreases were significantly greater in females than in males.
- The reduction in NTD cases during the post-FA period inversely mirrors the increase in plasma folate concentrations among pregnant women.
- Limitations of our study include that covering small regions and lacking of detailed subtypes of spina bifida.

For peer review only

INTRODUCTION

The prevalence of many types of birth defects appears to differ by sex, with the majority of them being more prevalent among males, [1 2]including orofacial cleft (cleft lip with or without cleft palate), urinary system (hypospadias, hydronephrosis), and gastrointestinal tract (diaphragmatic hernia). However, some types of neural tube defects (NTDs) occur more frequently in females (e.g., isolated cases of cranial defects, such as anencephaly).[3, 4]Females tend to have anencephaly more often than males, while craniorachischisis and spina bifida involving the higher spine may also show a preponderance among females. [4] By contrast, there tends to be a higher proportion of males than females with spina bifida affecting the lower spine. [4] There are thought to be multiple initiation sites in human neural tube closure, [5] as described in other mammalian embryos. Neural tube closure occurs during weeks 3–4 of human gestation and the sex of the embryo is differentially associated with a lack of closure of specific areas of the neural tube. [5]

Female predisposition to anencephaly is also observed among a number of mouse models, [3, 6] but the mechanism underlying this has not been established. In mice, female embryos tend to be developmentally delayed compared to their male littermates at neurulation stages, leading to the hypothesis that the longer duration of cranial neural tube closure would lead to greater susceptibility to NTD-causing insult. However, going against this hypothesis, the delay in development may occur prior to neural tube closure, with female and male embryos taking the same time to complete cranial neurulation. [7]Other suggested mechanisms include sex differences in genetic risk, epigenetic factors, a differential rate of fetal loss, and susceptibility to environmental influences. [8]

Among environmental factors, periconceptional supplementation with folic acid (FA) has been shown to dramatically reduce the risk for NTDs in offspring [9]and conversely, suboptimal maternal folate status is a risk factor for NTDs.[10] Although a large number

For peer review only - http://bmjopen.bmj.com/site/about/guidelines.xhtml

of studies have investigated the preventive effects of FA on NTDs, the influence of maternal FA supplementation on sex-associated risk for NTDs and their subtypes is unclear. Some studies have lacked data on the type of NTDs or sex, [11] while others have not found significant differences among subtypes of NTD [12]. In these studies the apparent protective effects of FA does not seem toact preferentially in one region of the neural tube and there appears to be a general reduction in the occurrence of human NTDs.[13] In a study in Mexico in which weekly administration of 5 mg FA was associated with a 50% reduction in the incidence of NTDs, the main effect was on spina bifida, with a higher reduction in female cases.[8] In several countriesfolic acid fortification (FAF) of food has been associated with a decreased frequency of spina bifida and anencephaly on a population level. [14 15]. A recent study from South America showed that prevalence of NTDs, particularly anencephaly and cervico-thoracic spina bifida, showed a greater reduction rate in females than in males after FAF resulting in a change of the sex ratio of infants with NTDs [16].

In 2009, the Ministry of Health of China initiated a nationwide FA supplementation program for the purpose of further decreasing the occurrence of NTDs in the country. [17] Women with a household registration and planning to become pregnant are eligible to obtain FA supplements free of charge through the maternal health care system. In a recent study, we showed that birth prevalence of NTDs in the five counties of Shanxi Province in northern China dropped dramatically, from 118.9/10,000 in 2000 to 31.5/10,000 in 2014. [18]In the present study, we examined the trends in NTD prevalence by sex in five counties of Shanxi Province, China, from 2003 to 2016, encompassing the introduction of a massive FA supplementation campaign. Characterization of the sex distribution of NTDs may be helpful to better understand NTD development in association with FA supplementation.

MATERIALS AND METHODS

Birth defect surveillance

Five counties in Shanxi Province (Pingding, Xiyang, Taigu, Zezhou, and Shouyang)

For peer review only - http://bmjopen.bmj.com/site/about/guidelines.xhtml

were included in a population-based birth defects surveillance system in the current study. The surveillance system was similar to a previously reported system, created to meet the primary objective of monitoring the prevalence of major structural birth defects including NTDs.[19] A national FA supplementation program was initiated in China in 2009; therefore, in the current study we collected data at two time points: before (2003–2004) and after (2011–2016) the start of the supplementation program. All pregnant women who resided in the study area for more than 1 year during the period were monitored. All live births (28 or more complete gestational weeks), all stillbirths of at least 20 weeks' gestational age, and pregnancy terminations at any gestational age following the prenatal diagnosis of NTDs were included. Photographs of every suspected case were obtained. NTD diagnosis was conducted by local specialists in maternal-fetal medicine, and photographs were sent to a pediatrician at Peking University for confirmation.The study protocol was reviewed and approved by the Institutional Review Board of Peking University.

Statistical analysis

Theprevalence of NTDs by infant sex was analyzed separately for open NTDs (anencephaly, spina bifida) [20] and encephalocele. The denominator was the total number of all births and pregnancy that reached 28 or more complete gestational weeks by sex; the numerator was the number of NTDs cases regardless of gestational age by sex. The prevalence of NTDs by sex and subtype were compared using Chi-square tests. A difference in differences (DID) model was applied to compare sex differences between the time periods. [21] A two-tailed $p \leq 0.05$ was considered to indicate statistically significant differences. All statistical analyses were performed using the SPSS package version 20.0 (SPSS Inc., Chicago, USA).

Patient and Public Involvement

Our main analysis was based on the birth defects surveillance system; the information was anonymized. Patients and or public were not involved.

RESULTS

For peer review only - http://bmjopen.bmj.com/site/about/guidelines.xhtml

Prevalence Before FA Supplementation Program

The prevalence of NTDs by subtype and sex are shown in Table 1. Before the program, a total of 25,249 births and 341 cases of NTDs (156 males, 185 females) were recorded in the system. The overall prevalence of NTDs for both sexes was 135.1/10,000, comprising 118.6/10,000 among males and 152.9/10,000 among females. All subtypes except spina bifida were more prevalent among females with a male-to-female RR of 0.60 (95% CI, 0.45–0.80) and 0.79 (95% CI, 0.77–0.81) for total NTDs and open NTDs, respectively (Table 1).

Table 1. Prevalence Rates of NTDs (per 10,000 births) by Type and Sex in Shanxi Province, China, in 2003–2004 and 2011–2016

NTDs type	Male			Female			M:F rate ratio		
	Births	Cases	Rate	Births	Cases	Rate	RR	95% CI	
2003–2004 (Pre-FA supplementation)									
	13 150			12 099					
Open NTDs		145	110.3		168	138.9	0.79	0.77	0.81
Anencephaly		65	49.4		94	77.7	0.64	0.61	0.67
Spina bifida		80	60.8		74	61.2	0.99	0.94	1.04
Encephalocele		11	8.4		17	14.1	0.60	0.45	0.80
Total NTDs		156	118.6		185	152.9	0.78	0.76	0.80
2011–2016 (Post-FA supplementation)									
	43 538			40 458					
Open NTDs		160	36.7		158	39.1	0.94	0.92	0.96
Anencephaly		64	14.7		77	19.0	0.77	0.73	0.81
Spina bifida		96	22.0		81	20.0	1.10	1.05	1.15
Encephalocele		17	3.9		21	5.2	0.75	0.61	0.92
Total NTDs		177	40.7		179	44.2	0.92	0.90	0.94

Prevalence During FA Supplementation Program

After the initiation of the program, a total of 82,258 births and 356 cases of NTDs (177 males, 179 females) were recorded in the system. The overall prevalence for both sexes was 43.3/10,000, being 40.7/10,000 among males and 44.2/10,000 among females. Hence, in sum, NTDs were less prevalent among males than females (RR, 0.92; 95% CI, 0.90–0.94), as was anencephaly (RR, 0.77; 95% CI, 0.73–0.81) and encephalocele (RR, 0.75; 95% CI, 0.61–0.92), while spina bifida showed male predominance (RR, 1.10; 95% CI, 1.05–1.15) (Table 1).

For peer review only - http://bmjopen.bmj.com/site/about/guidelines.xhtml

Differences in Changes in Prevalence Rates of NTDs

After the supplementation period, the prevalence of all types of NTDs was lower in both male and female fetuses. However, there was a significant sex difference in the magnitude of reduction, being more pronounced among females, especially for open NTDs including anencephaly (*p*<0.01). The overall prevalence of NTDs decreased by 78/10,000 in males and 108.7/10,000 in females in absolute terms; anencephaly decreased by 34.7% in males and 58.7% in females in relative term (Table 2).

Table 2. Differences in Changes in Prevalence Rates of NTDs (per 10,000 births) by Type and Sex in Shanxi Province, China, in 2003–2004 and 2011–2016

Types	Male rate		Female rate		Female-Male DID Value	P
	Absolute	Relative	Absolute	Relative		
**Open NTDs**	73.5	66.7	99.8	71.9	26.3	0.001
Anencephaly	34.7	70.3	58.7	75.5	23.9	0.001
Spina bifida	38.8	63.8	41.1	67.3	2.4	0.765
**Encephalocele**	4.5	53.3	8.9	63.1	4.4	0.213
**Total NTDs**	78.0	65.7	108.7	71.1	30.7	0.007

Note:

1. Absolute rate of decrease in prevalence = Prevalence of group before folic acid supplementation – Prevalence of group after folic acid supplementation
2. Relative rate of decrease in prevalence = Absolute rate of decrease in prevalence/Prevalence of group before folic acid supplementation

For peer review only

DISCUSSION

This quasi-experimental population-based study examined whether introduction of population-wide FA supplementation was associated with changes in the sex ratio for NTDs prevalence in northern China, as a means to understand whether the preventive effects of FA supplementation differ by sex. We found that, after the introduction of the national FA supplementation campaign, the prevalence of total NTDs and open NTDs (including anencephaly, spina bifida) decreased among both sexes. However, the magnitude of decline was significantly greater among females than males.

The prevalence of NTDs in Shanxi Province in northern China is historically high, being 105.5 per 10,000 births in the late 1980s [22] and 138 per 10,000 births in 2003.[23] In 2009, the Ministry of Health of China initiated a nationwide FA supplementation

For peer review only - http://bmjopen.bmj.com/site/about/guidelines.xhtml

program for the purpose of further decreasing the occurrence of NTDs in the country.[17] Women with a household registration and planning to become pregnant are eligible to obtain FA supplements free of charge through the maternal health care system. In a recent study, we showed that birth prevalence of NTDs in the five counties of Shanxi Province in northern China dropped dramatically, from 118.9/10,000 in 2000 to 31.5/10,000 in 2014.[18] However, differences between the sexes among this population have rarely been examined. One previous study reported a female predominance among anencephaly cases (male-to-female relative risk [RR], 0.49; 95% confidence interval [CI], 0.30–0.79) but not among spina bifida (RR, 0.90; 95% CI, 0.55–1.45) or encephalocele (RR, 1.03; 95% CI, 0.40–2.69) cases in 2006;[23] however, that study was conducted before the national FA supplementation program and how the NTD prevalence has changed in each sex in association with the program remains unknown.

Since 2009, the nationwide FA supplementation program has provided FA supplements, free of charge, to all women who have a rural registration and who plan to become pregnant. As we previously reported, the prevalence of NTDs was dramatically lower in 2012–2014 than in 2009 in Shanxi Province. This decrease may be partially attributable to the program; however, this decreasing trend has not continued since 2014. In fact, the prevalence in the same area was higher in 2015–2016 (32.8 per 10,000 births) than in 2014 (31.5 per 10,000 births). Moreover, the prevalence of NTDs in this area remains high when compared to the rates in the USA,[15] Canada,[24] Germany,[25] England, Wales,[26] and Western Australia.[27] The compliance of women taking the folic acid pills may be attributable to the high prevalence. A previous study showed the percentage of folic acid supplementation before the last menstrual period increased only from 30% to 43% and the supplementation adherence of ⩾8 days/10 days showed a decrease.[28] Other reasons may need further study.

In addition to the NTD prevalence data, our previous cross-sectional surveys on the

For peer review only - http://bmjopen.bmj.com/site/about/guidelines.xhtml

use of FA and blood folate concentration among pregnant women provide external evidence to test the correlation between folate status and NTDs. Those two surveys took place in the same area, one before the FA program started (2003–2004)[29] and the other after it started (2011–2012).[30]Detailed descriptions of the two studies have already been published.[30 31] Briefly, a face-to-face interview with a structured questionnaire including information on folic acid supplementationwas conducted among women who was in their late first trimester or early second trimester of pregnancy. A 5-ml non fasting blood sample was collected at the time of recruitment and plasma levels of folate were determined using a microbiological assay.[32]

Our previous cross-sectional study showed that the proportion of people taking FA supplements increased from 9.2% in 2003–2004 [33] to 66.3% in 2011–2012[30]. Comparison of population data on blood folate concentration among pregnant women and NTD prevalence data indicated a clear trend: plasma folate concentration increased from 10.4 nmol/L in 2003–2004[33] to 33.4 nmol/L in 2011–2012[22] while the prevalence of NTDs decreased from 117.8/10,000 births to 60.3/10,000 births, respectively[19]. Women who took FA supplements had significantly higher folate concentrations (median 41.9 nmol/L) than those who did not (13.2 nmol/L) in 2003–2004. In contrast, these values were 50.7 nmol/L and 17.3 nmol/L, respectively, in 2011–2012.The prevalence of NTDs was lower among both males and females after the program.However, the effect was not the same for males and females, being greater among females than males. For open NTDs, the decrease was 26.3/10,000 higher among females than males; for total NTDs (including encephalocele), the decrease was 30.7/10,000 higher among females than malesin current study.

Studies on mice have indicated that female embryos are more susceptible to NTDs than males in some, but not all, genetic models.[34]For example, there is a higher rate of cranial NTDs in mice carrying mutations in *Trp53*, [6]*Pax3*,or *Nf1*[35] but not *Gldc*.[36] Sex differences in NTD susceptibility in *Trp53* null mice may result from the presence of two X chromosomes and independent of the Y chromosome.[6] Given the association

For peer review only - http://bmjopen.bmj.com/site/about/guidelines.xhtml

between abnormal one-carbon metabolism or impaired methylation and NTDs, it has been hypothesized that the higher rate of cranial NTDs in females may be an epigenetic phenomenon. The demand for genomic DNA methylation is higher in females, which methylate most of the DNA in the large inactive X chromosome after every cell division. It is speculated that this may reduce the methylation capacity available for other key cellular needs.[3] In the *splotch* (Sp^{2H}; *Pax3* mutant) strain, female susceptibility to exencephaly is exacerbated by dietary folate deficiency to a greater extent than in males.[37] While the etiology of human NTDs is more complex than in single-gene models, if such a scenario occurs in some human NTDs, then it might be predicted that an overall increase in population-level folate status may have a greater effect on NTDs in females than in males. This is consistent with our findings for anencephaly rates.

Genetic and environmental factors may also interact to determine murine sex ratios, with female embryos being more susceptible to lethality. In the *splotch* strain, for example, arsenite treatment and advanced maternal age are both associated with higher rates of *in utero* death and NTDs.[38] Both factors result in elevated sex ratios (male:female) among litters, which suggests increased loss of female embryos by resorption. This effect is genotype-dependent such that a greater effect, with lower sex ratio, is noted as the number of *Sp*(*Pax3* mutant) alleles increases.

A study of human NTDs indicated an overall excess of affected females, with the overall M:F sex distribution of unaffected carrier or transmitting individuals was 0.64, and an even greater excess of female gene carriers, with a M:F ratio of 0.52, when only closely related relatives are counted. [39] To fully understand the genetic influences of NTDs, future studies are needed to investigate the importance of epigenetic factors andimprinting effects, methylation status, and folate supplementation, as well as the gender of the child in altering NTD risk.

For peer review only - http://bmjopen.bmj.com/site/about/guidelines.xhtml

In fact, the larger decline in NTD prevalence among females is somewhat expected given that it was higher in the pre-supplementation time period. The difference between sexes is less pronounced is consistent with prevalences beginning to converge toward the prevalence of NTDs that are not preventable by folic acid.

Our data are from a population-based surveillance system, which covered all live births (28 or more complete gestational weeks), and NTD cases from pregnancies of any gestational age. This could give a more precise estimation of NTD rate than hospital-based surveillance systems that include only NTD cases in pregnancies of 28 gestational weeks or greater in China. [40]

Three main limitations of our study should be mentioned. First, we included data from only five counties, and the results may not be generalizable to the entire province or country. Second, we did not include the detailed subtypes of spina bifida, such as high-level and low-level lesions, which may show different patterns. A future study may be useful to address this question. Third, as an ecological design in which some confounding factors might affect our results, the massive FA supplementation program was the only major factor that may affect the population rate of NTDs in the study. Improvement in living standards and prenatal diagnosis may also impact the rate of NTDs, but these potential factors is not predicted to have adifferential effect on males and females.

In conclusion, the prevalence of NTDs decreased in both sexes after the implementation of a massive FA supplementation program, but to a greater degree (both total NTDs and open NTDs) in females.

Statement on ethical approval and data sharing:

Our study protocol was reviewed and approved by the Institutional Review Board of Peking University. Dr. Zhiwen Li took full responsibility to the data and data could be shared upon requirement.

For peer review only

Competing Interests: None declared.

Funding: This work was supported in part by the National Key Research and Development Program, Ministry of Science and Technology, P.R. China (grant No. 2016YFC1000500), National Natural Science Foundation of China (No. 81373014, No. 81511130088 and No. 81202265) and Medical Research Council (United Kingdom, N003713). NG is supported by Great Ormond Street Hospital Children's Charity.

For peer review only

References

1. Hay S. Sex differences in the incidence of certain congenital malformations: a review of the literature and some new data. *Teratology* 1971;4(3):277-86. doi: 10.1002/tera.1420040303

2. Michalski AM, Richardson SD, Browne ML, et al. Sex Ratios Among Infants with Birth Defects, National Birth Defects Prevention Study, 1997-2009. *Am J Med Genet A* 2015;167(5):1071-81. doi: 10.1002/ajmg.a.36865

3. Juriloff DM, Harris MJ. Hypothesis: the female excess in cranial neural tube defects reflects an epigenetic drag of the inactivating X chromosome on the molecular mechanisms of neural fold elevation. *Birth defects research Part A, Clinical and molecular teratology* 2012;94(10):849-55. doi: 10.1002/bdra.23036

4. Tennant PW, Samarasekera SD, Pless-Mulloli T, et al. Sex differences in the prevalence of congenital anomalies: a population-based study. *Birth defects research Part A, Clinical and molecular teratology* 2011;91(10):894-901. doi: 10.1002/bdra.22846 [published Online First: 2011/10/12]

5. Seller MJ. Sex, neural tube defects, and multisite closure of the human neural tube. *American journal of medical genetics* 1995;58(4):332-6. doi: 10.1002/ajmg.1320580406 [published Online First: 1995/09/25]

6. Chen X, Watkins R, Delot E, et al. Sex difference in neural tube defects in p53-null mice is caused by differences in the complement of X not Y genes. *Developmental neurobiology* 2008;68(2):265-73. doi: 10.1002/dneu.20581 [published Online First: 2007/11/16]

7. Brook FA, Estibeiro JP, Copp AJ. Female predisposition to cranial neural tube defects is not because of a difference between the sexes in the rate of embryonic growth or development during neurulation. *Journal of medical genetics* 1994;31(5):383-7. [published Online First: 1994/05/01]

8. Martinez de Villarreal LE, Arredondo P, Hernandez R, et al. Weekly administration of folic acid and epidemiology of neural tube defects. *Matern Child Health J* 2006;10(5):397-401. doi: 10.1007/s10995-006-0082-2

9. Berry RJ, Li Z, Erickson JD, et al. Prevention of neural-tube defects with folic acid in China. China-U.S. Collaborative Project for Neural Tube Defect Prevention. *N Engl J Med* 1999;341(20):1485-90. doi: 10.1056/NEJM199911113412001

10. Crider KS, Devine O, Hao L, et al. Population red blood cell folate concentrations for prevention of neural tube defects: Bayesian model. *BMJ* 2014;349:g4554. doi: 10.1136/bmj.g4554

11. Smithells RW, Sheppard S, Schorah CJ, et al. Apparent prevention of neural tube defects by periconceptional vitamin supplementation. 1981. *Int J Epidemiol* 2011;40(5):1146-54. doi: 10.1093/ije/dyr143

12. MRC Vitamin Study Research Group. Prevention of neural tube defects: results of the Medical Research Council Vitamin Study. . *Lancet* 1991;338(8760):131-7.

13. Seller MJ. Multi-site neural tube closure in humans and maternal folate supplementation. *American journal of medical genetics* 1995;58(3):222-4. doi: 10.1002/ajmg.1320580305

14. Honein MA, Paulozzi LJ, Mathews TJ, et al. Impact of folic acid fortification of the US food supply on the occurrence of neural tube defects. *JAMA* 2001;285(23):2981-6.

15. Williams J, Mai CT, Mulinare J, et al. Updated estimates of neural tube defects prevented by mandatory folic Acid fortification - United States, 1995-2011. *MMWR Morb Mortal Wkly Rep* 2015;64(1):1-5.

For peer review only - http://bmjopen.bmj.com/site/about/guidelines.xhtml

16. Poletta FA, Rittler M, Saleme C, et al. Neural tube defects: Sex ratio changes after fortification with folic acid. *PLoS One* 2018;13(3):e0193127. doi: 10.1371/journal.pone.0193127
17. Ren AG. Prevention of neural tube defects with folic acid: The Chinese experience. *World J Clin Pediatr* 2015;4(3):41-4. doi: 10.5409/wjcp.v4.i3.41
18. Liu J, Zhang L, Li Z, et al. Prevalence and trend of neural tube defects in five counties in Shanxi province of Northern China, 2000 to 2014. *Birth defects research Part A, Clinical and molecular teratology* 2016;106(4):267-74. doi: 10.1002/bdra.23486
19. Li S, Moore CA, Li Z, et al. A population-based birth defects surveillance system in the People's Republic of China. *Paediatric and perinatal epidemiology* 2003;17(3):287-93.
20. McComb JG. A practical clinical classification of spinal neural tube defects. *Child's nervous system : ChNS : official journal of the International Society for Pediatric Neurosurgery* 2015;31(10):1641-57. doi: 10.1007/s00381-015-2845-9 [published Online First: 2015/09/10]
21. Ashenfelter O, Card D. Using the Longitudinal Structure of Earnings to Estimate the Effect of Training-Programs. *Rev Econ Stat* 1985;67(4):648-60. doi: Doi 10.2307/1924810
22. Xiao KZ, Zhang ZY, Su YM, et al. Central-Nervous-System Congenital-Malformations, Especially Neural-Tube Defects in 29 Provinces, Metropolitan Cities and Autonomous Regions of China - Chinese-Birth-Defects-Monitoring-Program. *International Journal of Epidemiology* 1990;19(4):978-82.
23. Li Z, Ren A, Zhang L, et al. Extremely high prevalence of neural tube defects in a 4-county area in Shanxi Province, China. *Birth defects research Part A, Clinical and molecular teratology* 2006;76(4):237-40. doi: 10.1002/bdra.20248
24. De Wals P, Tairou F, Van Allen MI, et al. Reduction in neural-tube defects after folic acid fortification in Canada. *N Engl J Med* 2007;357(2):135-42. doi: 10.1056/NEJMoa067103
25. Nasri K, Ben Fradj MK, Hamdi T, et al. Epidemiology of neural tube defect subtypes in Tunisia, 1991-2011. *Pathol Res Pract* 2014;210(12):944-52. doi: 10.1016/j.prp.2014.06.027
26. Morris JK, Wald NJ. Prevalence of neural tube defect pregnancies in England and Wales from 1964 to 2004. *J Med Screen* 2007;14(2):55-9. doi: 10.1258/096914107781261945
27. Bower C, D'Antoine H, Stanley FJ. Neural Tube Defects in Australia: Trends in Encephaloceles and Other Neural Tube Defects Before and After Promotion of Folic Acid Supplementation and Voluntary Food Fortification. *Birth Defects Res A* 2009;85(4):269-73. doi: 10.1002/bdra.20536
28. Liu JF, Jin L, Meng QQ, et al. Changes in folic acid supplementation behaviour among women of reproductive age after the implementation of a massive supplementation programme in China. *Public health nutrition* 2015;18(4):582-88. doi: 10.1017/S1368980014000950
29. Ren A, Zhang L, Hao L, et al. Comparison of blood folate levels among pregnant Chinese women in areas with high and low prevalence of neural tube defects. *Public health nutrition* 2007;10(8):762-68. doi: 10.1017/S1368980007246786
30. Liu J, Gao L, Zhang Y, et al. Plasma folate levels in early to mid pregnancy after a nation-wide folic acid supplementation program in areas with high and low prevalence of neural tube defects in China. *Birth defects research Part A, Clinical and molecular teratology* 2015;103(6):501-8. doi: 10.1002/bdra.23368 [published Online First: 2015/03/27]
31. Ren AG, Zhang L, Li ZW, et al. Awareness and use of folic acid, and blood folate concentrations among pregnant women in northern China - An area with a high prevalence of neural tube defects. *Reprod Toxicol* 2006;22(3):431-36. doi: 10.1016/j.reprotox.2006.02.003
32. O'Broin S, Kelleher B. Microbiological assay on microtitre plates of folate in serum and red cells. *J*

For peer review only - http://bmjopen.bmj.com/site/about/guidelines.xhtml

Clin Pathol 1992;45(4):344-7.
33. Zhang L, Ren A, Li Z, et al. Folate concentrations and folic acid supplementation among women in their first trimester of pregnancy in a rural area with a high prevalence of neural tube defects in Shanxi, China. *Birth defects research Part A, Clinical and molecular teratology* 2006;76(6):461-6. doi: 10.1002/bdra.20271
34. Copp AJ, Stanier P, Greene ND. Neural tube defects: recent advances, unsolved questions, and controversies. *The Lancet Neurology* 2013;12(8):799-810. doi: 10.1016/s1474-4422(13)70110-8 [published Online First: 2013/06/25]
35. Lakkis MM, Golden JA, O'Shea KS, et al. Neurofibromin deficiency in mice causes exencephaly and is a modifier for Splotch neural tube defects. *Dev Biol* 1999;212(1):80-92. doi: 10.1006/dbio.1999.9327
36. Pai YJ, Leung KY, Savery D, et al. Glycine decarboxylase deficiency causes neural tube defects and features of non-ketotic hyperglycinemia in mice. *Nat Commun* 2015;6:6388. doi: 10.1038/ncomms7388
37. Burren KA, Savery D, Massa V, et al. Gene-environment interactions in the causation of neural tube defects: folate deficiency increases susceptibility conferred by loss of Pax3 function. *Hum Mol Genet* 2008;17(23):3675-85. doi: 10.1093/hmg/ddn262
38. Martin LJ, Machado AF, Loza MA, et al. Effect of arsenite, maternal age, and embryonic sex on spina bifida, exencephaly, and resorption rates in the splotch mouse. *Birth defects research Part A, Clinical and molecular teratology* 2003;67(4):231-9. doi: 10.1002/bdra.10006 [published Online First: 2003/07/12]
39. Deak KL, Siegel DG, George TM, et al. Further evidence for a maternal genetic effect and a sex-influenced effect contributing to risk for human neural tube defects. *Birth defects research Part A, Clinical and molecular teratology* 2008;82(10):662-9. doi: 10.1002/bdra.20511 [published Online First: 2008/10/22]
40. Li K, He XB, Zhang L, et al. [Impact of pregnancy terminations before 28 weeks of gestation on the overall prevalence of neural tube defects in two counties of Shanxi province]. Zhonghua liu xing bing xue za zhi = Zhonghua liuxingbingxue zazhi 2012;33(5):509-12. [published Online First: 2012/08/14]

For peer review only

For peer review only - http://bmjopen.bmj.com/site/about/guidelines.xhtml

STROBE Statement—Checklist of items that should be included in reports of ***cross-sectional studies***

Pages		Item No	Recommendation
1-2	**Title and abstract**	1	(*a*) Indicate the study's design with a commonly used term in the title or the abstract
			(*b*) Provide in the abstract an informative and balanced summary of what was done and what was found
	**Introduction**		
4-5	Background/rationale	2	Explain the scientific background and rationale for the investigation being reported
5	Objectives	3	State specific objectives, including any prespecified hypotheses
	**Methods**		
6	Study design	4	Present key elements of study design early in the paper
5-6	Setting	5	Describe the setting, locations, and relevant dates, including periods of recruitment, exposure, follow-up, and data collection
6	Participants	6	(*a*) Give the eligibility criteria, and the sources and methods of selection of participants
6	Variables	7	Clearly define all outcomes, exposures, predictors, potential confounders, and effect modifiers. Give diagnostic criteria, if applicable
6	Data sources/ measurement	8*	For each variable of interest, give sources of data and details of methods of assessment (measurement). Describe comparability of assessment methods if there is more than one group
6	Bias	9	Describe any efforts to address potential sources of bias
6	Study size	10	Explain how the study size was arrived at
6	Quantitative variables	11	Explain how quantitative variables were handled in the analyses. If applicable, describe which groupings were chosen and why
7	Statistical methods	12	(*a*) Describe all statistical methods, including those used to control for confounding
7			(*b*) Describe any methods used to examine subgroups and interactions
Not applicable			(*c*) Explain how missing data were addressed
Not applicable			(*d*) If applicable, describe analytical methods taking account of sampling strategy
Not applicable			(*e*) Describe any sensitivity analyses
	**Results**		
7-8	Participants	13*	(a) Report numbers of individuals at each stage of study—eg numbers potentially eligible, examined for eligibility, confirmed eligible, included in the study, completing follow-up, and analysed
Not applicable			(b) Give reasons for non-participation at each stage
Not applicable			(c) Consider use of a flow diagram
7-8	Descriptive data	14*	(a) Give characteristics of study participants (eg demographic, clinical, social) and information on exposures and potential confounders
Not			(b) Indicate number of participants with missing data for each variable

For peer review only

applicable			of interest
7-9	Outcome data	15*	Report numbers of outcome events or summary measures
7-9	Main results	16	(*a*) Give unadjusted estimates and, if applicable, confounder-adjusted estimates and their precision (eg, 95% confidence interval). Make clear which confounders were adjusted for and why they were included
7-9			(*b*) Report category boundaries when continuous variables were categorized
7-9			(*c*) If relevant, consider translating estimates of relative risk into absolute risk for a meaningful time period
8-9	Other analyses	17	Report other analyses done—eg analyses of subgroups and interactions, and sensitivity analyses
	**Discussion**		
9	Key results	18	Summarise key results with reference to study objectives
12	Limitations	19	Discuss limitations of the study, taking into account sources of potential bias or imprecision. Discuss both direction and magnitude of any potential bias
9-12	Interpretation	20	Give a cautious overall interpretation of results considering objectives, limitations, multiplicity of analyses, results from similar studies, and other relevant evidence
11-12	Generalisability	21	Discuss the generalisability (external validity) of the study results
	**Other information**		
13	Funding	22	Give the source of funding and the role of the funders for the present study and, if applicable, for the original study on which the present article is based

*Give information separately for exposed and unexposed groups.

Note: An Explanation and Elaboration article discusses each checklist item and gives methodological background and published examples of transparent reporting. The STROBE checklist is best used in conjunction with this article (freely available on the Web sites of PLoS Medicine at http://www.plosmedicine.org/, Annals of Internal Medicine at http://www.annals.org/, and Epidemiology at http://www.epidem.com/). Information on the STROBE Initiative is available at www.strobe-statement.org.

For peer review only

For peer review only - http://bmjopen.bmj.com/site/about/guidelines.xhtml
